# Unveiling Structure-Dynamic Processes in Crystals of 1D Cd(II) Coordination Polymers during the Elastic Flexible Events

**DOI:** 10.1021/jacs.5c07191

**Published:** 2025-06-09

**Authors:** Marijana Đaković, Mateja Pisačić, Mladen Borovina, Ivan Kodrin, Adriana Kenđel, Tea Frey

**Affiliations:** Department of Chemistry, 117036 Faculty of Science, University of Zagreb, Zagreb 10000, Croatia

## Abstract

Flexibility, an intriguing yet far from being a commonly observed property of single crystals, is highly sought after as it enables crystal application in innovative technologies. Here, we report on anisotropically elastic single crystals of two novel 1D coordination polymers (CPs) featuring bridging halides (Cl (**1**); Br (**2**)) and pyrazinamide ligands that enabled the determination of the bending mechanism and exploration of their mechanical properties. The mechanism identified distinctly differs from the mechanisms of elastically flexible molecular crystals subjected to mechanical stress and 1D plastically deformable crystals under quasi-hydrostatic pressure, the mechanisms of reconfigurable crystals uncovered thus far. By mapping the structural modification across the bent crystal **1** using microfocus synchrotron radiation, we found that both the 1D structural spine (controlling the distance between the organic ligands) and the ligands themselves adapt to the changes in external conditions. While the spine expands through the modifications of the bridging angles, causing a consequent enlargement of the metal···metal distances as going from the interior to the exterior of the bend, the ligands rotate toward linearity with the bending face. In addition, the extent of changes along the two other crystal axes is brought into a connection with the relative energies of the weakest links and crystal stiffness in those directions.

## Graphical abstract

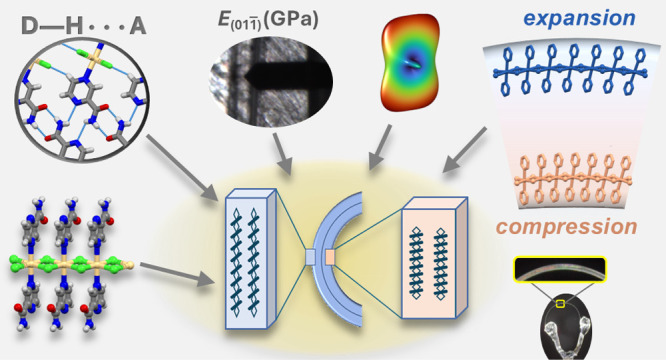

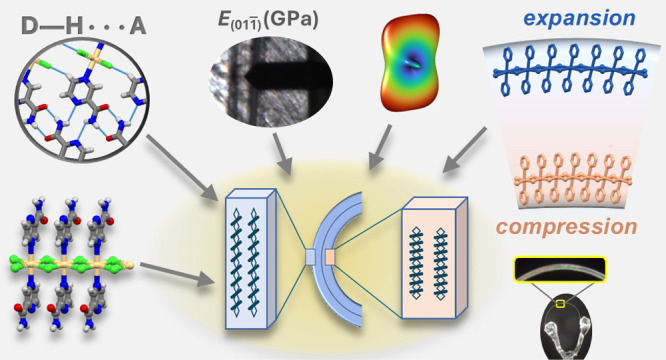

## Introduction

A significant portion of today’s technological advancement is founded on innovative and adaptive materials, where material elasticity plays a key role.^1–3^ The rise of crystal adaptronics^4,5^ has clearly demonstrated the vital importance of crystalline solids, positioning them as highly coveted materials for various advanced applications.^6–10^ Consequently, single-crystalline materials, endowed with adaptive properties and the ability to effectively regain their initial form, emerged at the forefront of solid-state and materials chemistry research.^11–16^ However, the successful integration of these materials into smart devices will undoubtedly hinge on our ability to precisely control their responses and develop a comprehensive understanding of their dynamic properties.

Reconfigurable crystals have so far demonstrated remarkable potential across a diverse array of advanced applications, spanning from optical waveguiding^17^ and (semi)­conductivity,^18–20^ to wearable devices^21^ and soft robotics.^22^ However, while the potential of these materials is undeniable, a deeper understanding of the fundamental mechanisms that bestow them with their remarkable flexibility and facilitate their seamless integration into sophisticated devices is far from being sufficiently understood.^23^

Current research on molecular mobilities of flexible crystals under elastic conditions is still in its early stages and remains quite limited while largely focusing on molecular crystals composed of discrete (0D) molecular building units.^24–26^ Consequently, all structural changes in the elastically strained crystals were attributed solely to adjustments made by the 0D molecular building units, which were considered entirely unchanged. To account for the differences in expansion and compression between the exterior and interior of a bent crystal, molecules were found to rotate and slip past each other while preserving their molecular geometry under external stress.^24–26^

However, the impact of mechanical pressure on molecular mobility within crystals composed of higher-dimensional building units (i.e., 1D or 2D), represents an intriguing and largely unexplored research domain. Understanding how these building blocks adjust to mechanical forces that induce alterations in the overall shape of a crystal can yield significant insights, as adaptive behavior is likely to involve transformations in molecular geometry. By concentrating our efforts on these dynamic processes, we can greatly improve our capability to engineer materials with tailored characteristics specifically designed for targeted applications.

To bridge the current gaps in our understanding of the connections between atomic-level adaptations and the reversible reshaping of crystals under mechanical stimuli, we are examining two novel 1D coordination compounds (CPs) of cadmium­(II) that utilize halides and pyrazinamide ligands (Figure 1a). The materials exhibit desired elastic behavior when subjected to external mechanical stimulation, providing a valuable opportunity to explore the atomic-level structural adaptations and uncover the bending mechanisms at play in the elastically strained 1D crystals.

**Figure 1:**
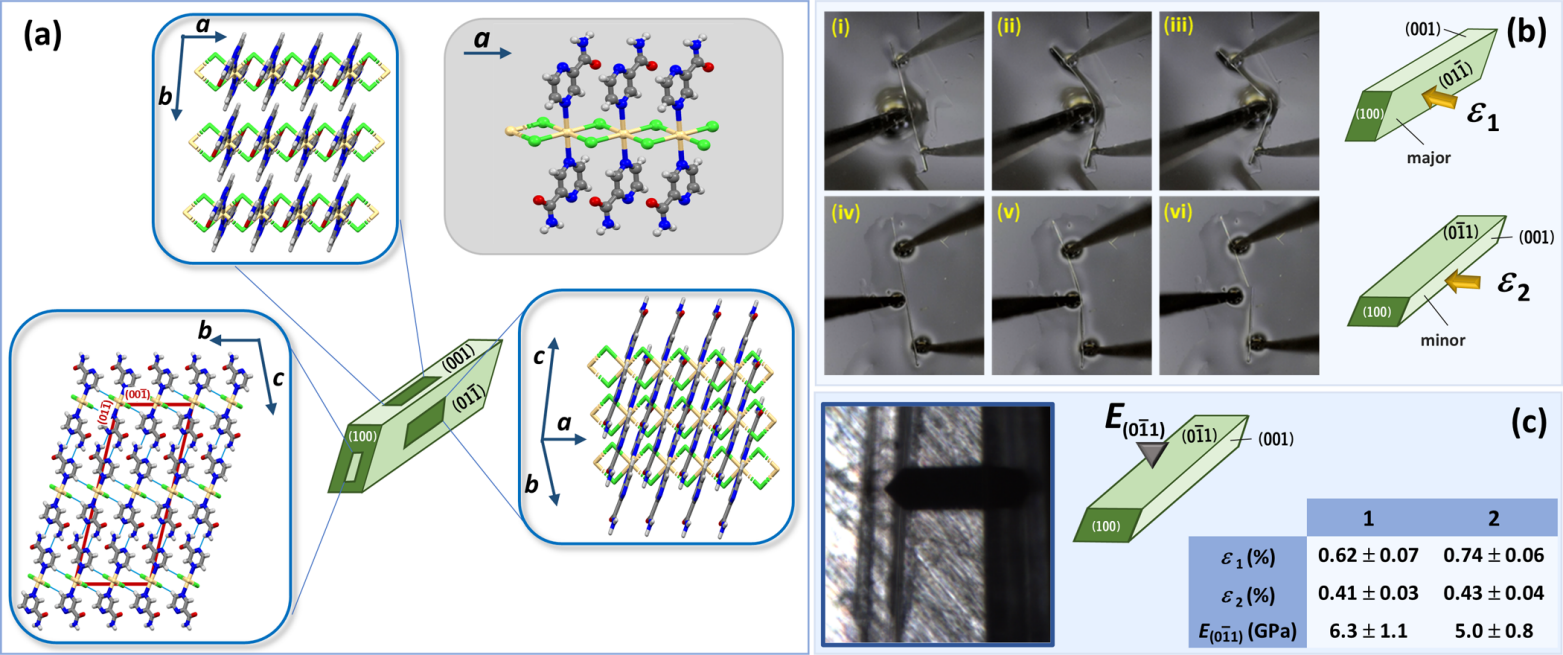
(a) Crystal morphology of [CdCl_2_(pza)_2_]_*n*_ (**1**) with face indices and crystal packing viewed in the planes of three crystal faces, (100), (001̅), and approx. (011̅). (b) Snapshots of the elastic bending of crystals of **1** by applying the mechanical stress on (011̅)/(01̅1) (top; i-iii) and (001̅)/(001) sets of crystal faces (bottom; iv-vi); crystals can be reversibly bent many times (i-ii; iv-v) as long as the critical radius is not exceeded (ii; v) when they eventually break (iii; vi). (c) Schematic representation of AFM nanoindentation measurements (left); Young’s moduli determined on (011̅)/(01̅1) crystal faces of undeformed crystals of **1** and **2**.

## Results and Discussion

Crystals of [CdCl_2_(pza)_2_]_*n*_ (**1**) and [CdBr_2_(pza)_2_]_*n*_ (**2**) (pza = pyrazinamide) were successfully grown using a layering technique from a water/ethanol solution and were formed as elongated needles measuring up to approximately 1 cm in length and 20 × 40 μm in a cross-section. They thus met the requisite quality and morphology criteria for crystallographic mapping studies and further evaluation of mechanical properties.

### Crystal Structure Description

Crystal structure determination revealed that **1** and **2** present isostructural packing arrangements in the space group *P*1̅. Both materials are composed of the intended 1D building units extending along the “short” crystallographic axis (i.e., *a* axis), with Cd­(II) centers doubly bridged by the halide anions, [M­(μ-X)_2_]_*n*_. The octahedral geometry around the Cd­(II) cations is completed by two pza ligands *trans*-oriented to each other. The 1D building units are mutually linked into a 3D network by three pairs of self-complementary hydrogen bonds, namely, C–H···Cl/Br, N–H···N, and N–H···O, all lying in the (111̅) plane (Tables S1–S3).

The optical microscopy of crystals **1** and **2** highlighted distinct differences between two pairs of prominent crystal faces aligned parallel to the longest crystal axis (i.e., [100]), while face indexing revealed these being (011̅)/(01̅1) and (001)/(001̅) as the major and minor crystal faces, respectively (Figure 1b; Figure S3).

### Mechanical Characterization

Mechanically stimulated flexibility of crystals **1** and **2** was assessed via a modified three-point bending experiment. Thin crystals were placed on a glass slide and submerged in a small amount of Paratone oil. Crystal morphology allowed for the evaluation of crystals’ flexibility in response to force stimulation in directions orthogonal to both the major and minor crystal faces. The force was exerted in a controlled manner (at a constant rate), enabling a comparison of the mechanical behaviors of the two materials, **1** and **2**.

Upon application of the force, crystals of **1** and **2** displayed notable elastic behavior in both directions; they effectively bent when pressure was exerted and subsequently returned to their original shape without any visible deterioration of the crystal shape once the force was released. However, they exhibited increased flexibility when the force was directed at the major (Movies 1 and 3) compared to the minor crystal face (Movies 2 and 4; Figure 2b). This observation reinforced previous findings related to elastic and elastoplastic 1D crystalline materials,^27^ highlighting the trait that has still not been commonly reported for flexible crystals. As a result, **1** and **2** can be effectively categorized as 2D anisotropically elastic materials, adding valuable insight into our understanding of crystal flexibility and its connectivity to structural arrangement.

**Figure 2:**
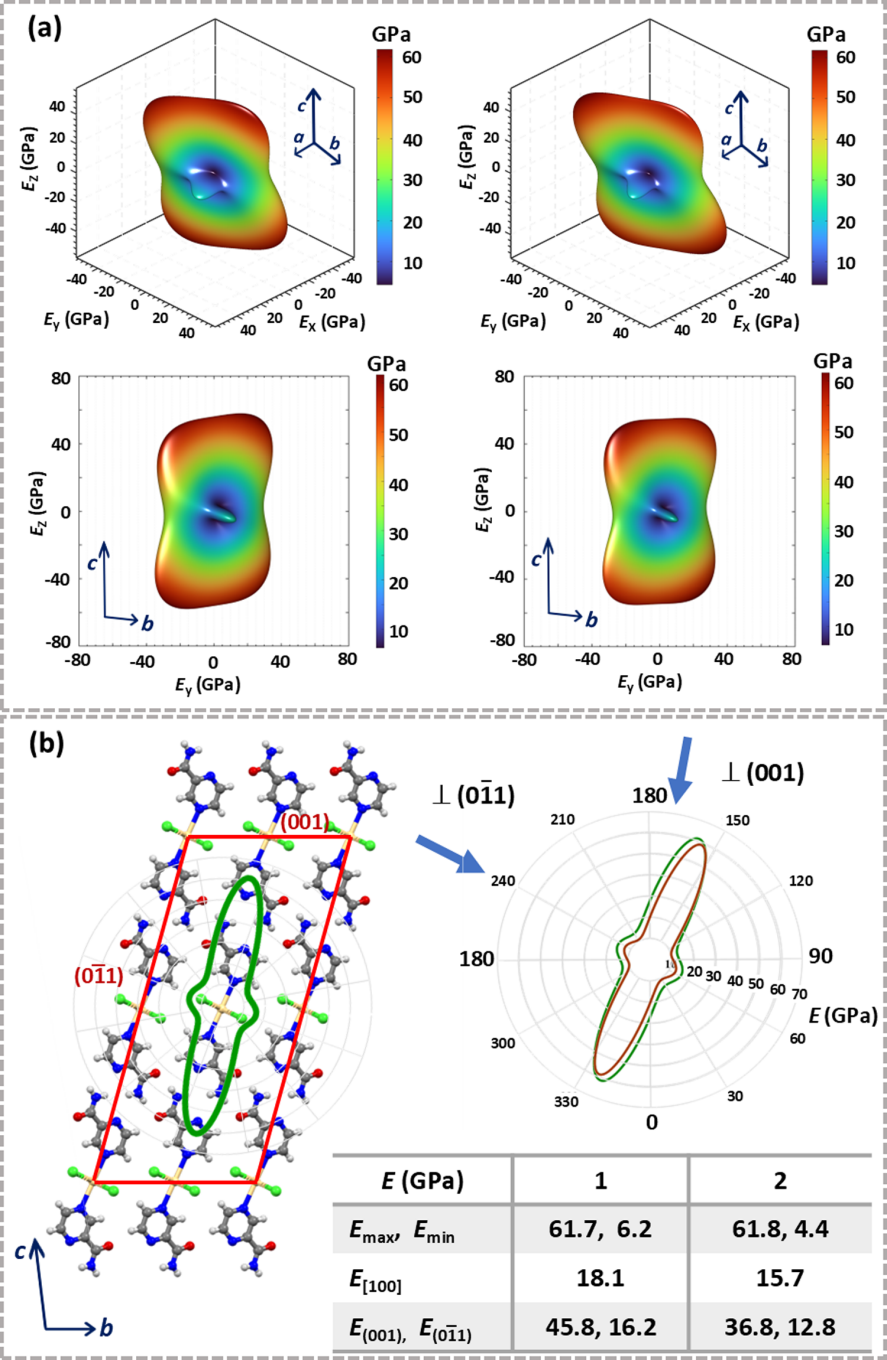
(a) 3D presentation of Young’s moduli (*E* in GPa) for **1** (left) and **2** (right) in an arbitrary direction (top) and down the crystallographic *a* axis (bottom). (b) Superposition of the 2D polar plot of *E* and crystal packing of **1** presented in the plane orthogonal to the *a* axis (left), and the overlay of 2D polar plots of *E* for **1** (green) and **2** (red) projected onto the plane orthogonal to the *a* axis (right).

The quantification of crystal behavior, utilizing Euler–Bernoulli’s beam bending theory,^28^ demonstrated distinct bending strain values (ε) for two directions, the force application on the major, ε_1_, and minor face, ε_2_, in both **1** and **2** (**1**: ε_1_ = 0.62 ± 0.07%, ε_2_ = 0.41 ± 0.03%; **2**: ε_1_ = 0.74 ± 0.06%, ε_2_ = 0.43 ± 0.04%; Figures S17–S19, Tables S12–S13). The values obtained in this study closely align with prior research regarding crystalline CPs, which report strain values ranging up to 1.3%.^29–31^ These findings are comparable to those documented for metals and metal alloys, which typically exhibit elastic strain of up to 1%.^32^ In contrast, organic molecular crystals (0D) tend to exhibit considerably larger strain values, with prior reports generally indicating a range between 2.4% and 5.6%,^33–35^ while in some rare instances, the strain can even approach 14%.^36,37^ This variation in strain behavior suggests potential unique mechanical properties associated with different material classes (0D vs 1D).

The mechanoflexible properties of crystals **1** and **2** were further examined using the AFM nanoindentation technique.^38^ The crystals were indented orthogonally to the major face, (01̅1), and the respective elastic modulus values (*E*_(01̅1)_) were retrieved (Figure 1c; Table S14). A subtle difference in *E* was detected, with **1** being slightly more resistant than **2** (**1**: *E*_(011̅)_ = 6.3 ± 1.1 GPa; **2**: *E*_(011̅)_ = 5.0 ± 0.8 GPa), indicating that consequences of even subtle structural differences, such as the exchange of a single atom (i.e., Cl for Br) can be discernible by the technique. The obtained *E* values correspond well to those reported for metal-containing 0D and 1D CP crystals, being in the range of 4.6–5.2 GPa;^39,40^ however, they are deceptively larger than those reported for organic crystals displaying a full elastic recovery, 0.15 ± 0.03 GPa.^41^

### DFT Computation of Mechanical Properties

The computational approach has proven as an effective method for rationalizing and predicting a diverse array of molecular properties, including mechanical responses of crystalline solids.^42–44^ However, reports on calculated elastic tensors for solids built up on weak intermolecular interactions remain scarce.^45–47^ In this work, we utilized computational techniques to elucidate the observed variations in the flexibility of our materials when subjected to external forces (for more details, see SI *Computational studies*).

The Young’s moduli (*E*) for **1** and **2**, computed at the PBE-D3/pob-TZVP-rev2 level of theory, are illustrated in Figure 2a. They clearly demonstrate a substantial anisotropy in *E* of both materials, highlighting a strong directional dependence on their mechanical properties. The analysis of *E* within the framework of crystal packing reveals that the directions exhibiting neither the highest (*E*_max_: 61.7 GPa (**1**), 61.8 GPa (**2**)) nor the lowest stiffness (*E*_min_: 6.2 GPa (**1**), 4.4 GPa (**2**)) are oriented along the main crystal axis, i.e., the *a* axis (Figure 2b). While the directions corresponding to *E*_min_ are inclined toward the spreading of the polymeric [Cd­(μ-X)_2_]_*n*_ chains (approximately at a 30° angle), the directions exhibiting *E*_max_ are situated in the plane containing the pza ligands (i.e., the (111̅) plane). In fact, there are two major directions associated with *E*_max_, and these align closely with (i) the directions of elongation of the Cd–N_pza_ and N–H···N bonds, namely the [011] direction, and (ii) orientations involving the pyrazine rings and N–H···O hydrogen bonds, approximately the [32̅1] direction. In contrast to *E*_max_, which presented almost identical values for the two materials (*E*_max_(**1**) ≈ *E*_max_(**2**)), *E*_min_ reveals a small yet perceptible difference (*E*_min_(**1**) > *E*_min_(**2**)).

Moreover, noticeable differences in elastic modulus are also evident in main crystal directions, along the longest crystal axis, [100], and in the directions orthogonal to the bending faces, (011̅/01̅1) and (001/001̅). In every instance, the *E* values were found to be greater for material **1** compared to material **2** (*E*_[100]_/*E*_(001)_/*E*_(01̅1)_: 18.1 GPa/45.8 GPa/16.2 GPa (**1**); 15.7 GPa/36.8 GPa/12.8 GPa (**2**); Figure 2b, inset), presenting the differences that parallel those experimentally probed on the (011̅)/(01̅1) faces (i.e., being approximately 20%).^48^

Furthermore, the analysis of *E* in the context of crystal packing reveals a clear connection between variances in elastic constants and intermolecular interactions. The overlay of polar plots (Figure 2b) reveals small but perceptible distinctions (*E*_**1**_ > *E*_**2**_) in all directions influenced by supramolecular interactions involving halides, C–H···Cl/Br. In contrast, in directions that are largely unaffected by these interactions, the variances in *E* are much smaller or even negligible. Although the existing differences between the two materials are relatively minor, they still effectively demonstrate the impact that intermolecular interactions have on the mechanical properties of these materials.

### Synchrotron Microfocus X-ray Diffraction (μ-SCXRD)

Gaining insight into the interplay of collective movements that alter materials’ macroscopic shape while preserving their structural integrity is crucial for realizing their full potential in practical settings. To understand the atomic-level changes during the elastic bending of our 1D materials, crystals of **1** were examined by synchrotron microfocus diffraction experiments (μ-SCXRD). The method established previously and employed for the determination of the mechanisms of three derivatives of [Cu­(acac)_2_] ^24,25,49^ and a cocrystal of caffeine and 4-chloro-3-nitrobenzoic acid^26^ was adopted in this study with necessary modifications (to account for the heavy atom presence; for details see *Synchrotron microfocus SCXRD* section in SI). The deformations in the main crystal directions, as well as fine structural adaptation, were determined through the mapping of the unit cell parameters. For that purpose, a crystal of **1** of sufficient size was bent down the major face, (011̅/01̅1), secured on a glass holder in a bent shape, and profiled across the bend (unfortunately, crystals of **2** did not allow analogues experiment; for details, see SI). Additionally, structures of **1** and **2** were determined at both the convex and concave sides of the apex of the bent crystals (using larger data sets; Tables S4–S5, Figure S8).

The mapping results of **1** demonstrated that the crystallographic *a* axis, which aligns with the main crystal axis, expands on the convex and compresses on the concave side of the bent crystal (Figure 3a,b). This is generally expected for the elastically flexible materials in a bent form and established for the four μ-SCXRD-characterized elastically flexible *molecular* crystals.^24–26^ Furthermore, the two other directions orthogonal to the major crystal axis, which indicate the deformation associated with the major and minor bending faces, present the opposite trend, expanding in the interior and compressing on the exterior of the bend (Figure 3b *top*), which also aligns with previous findings.^24,26^ However, despite the analogous trends in the deformations along the three relevant directions, more intricate structural mobility was observed in **1** than in the four molecular crystals. For molecular crystals, molecules themselves (i.e., 0D building units) were found to be unchanged throughout the sample, consequently constraining the crystal bending to materialize solely through movements such as reversible rotation and sliding of molecules past each other so as to retain the constant interplanar distance. In contrast, in **1**, the changes in the major crystallographic axis led to changes in the building units themselves (i.e., 1D polymeric chains), which in turn resulted in changes in the Cd···Cd *intra*chain distances, being enlarged and decreased on the convex and concave parts of the crystal, respectively. In addition, the opposite trends in changes in the other two directions in **1** affect the *inter*chain distances, bringing the elongated CP building units closer to each other at the outer and pushing them further apart at the inner arc of the crystal.

**Figure 3:**
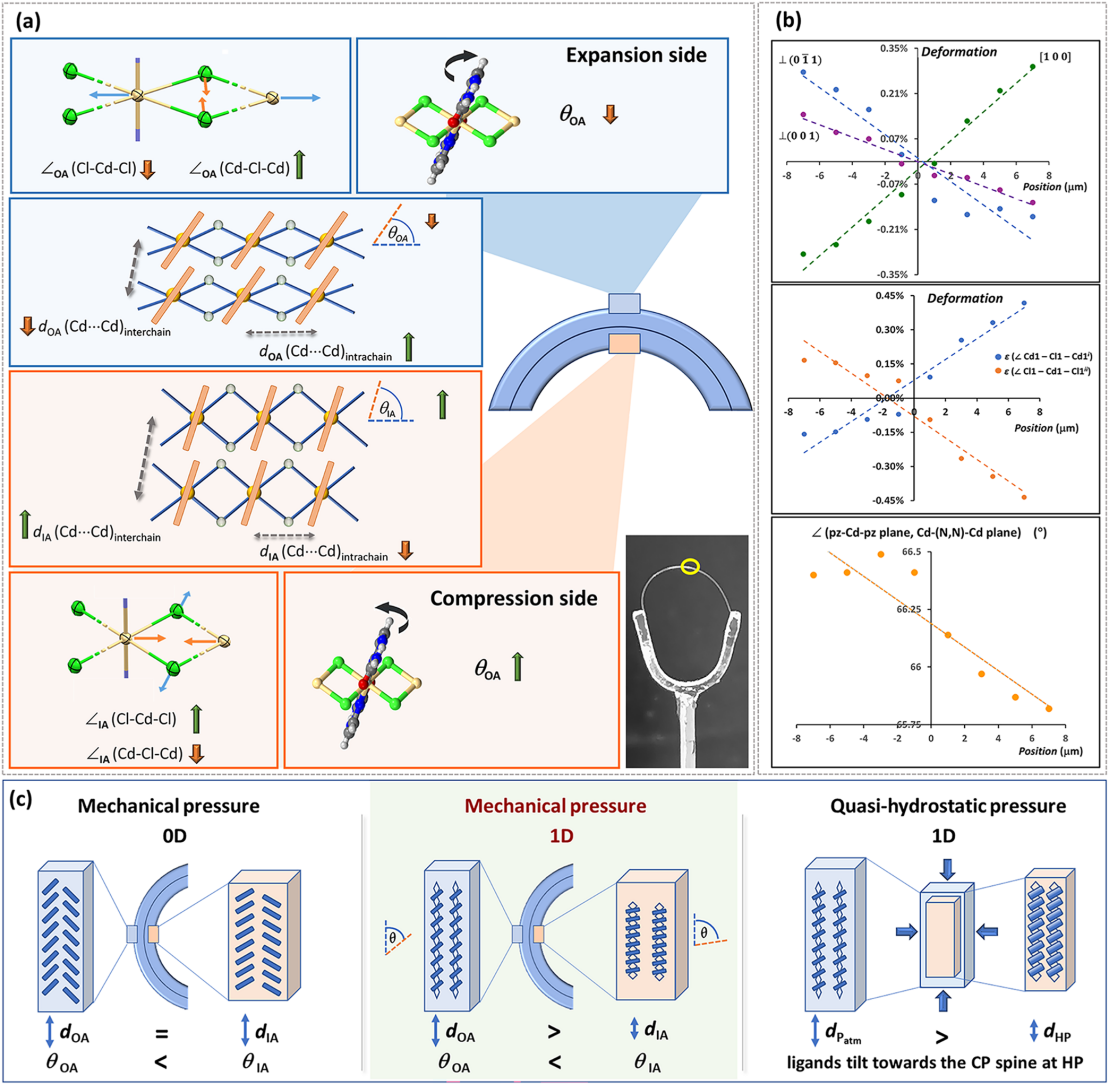
(a) The schematic representation of structural changes observed on two sides of the crystal bend of **1**, expansion (outer arc, OA; marked with blue frames and shading) and compression (inner arc, IA; marked with orange frames and shading), including a photograph of the bent crystal examined in the synchrotron μ-SCXRD mapping experiment. On the expansion side, the [Cd­(μ-Cl)_2_]_*n*_ polymeric chains are extended, as evidenced by the increase in the *intra*chain Cd···Cd distance (*d*_OA_(Cd···Cd)_*intra*chain_) and simultaneous alterations in the *intra*chain Cd–Cl–Cd and Cl–Cd–Cl angles (the decrease in angle ∠_OA_(Cl–Cd–Cl) and increase in angle ∠_OA_(Cd–Cl–Cd)). This extension of the CP chains is accompanied by a reduction in the *inter*chain Cd···Cd distances (*d*_OA_(Cd···Cd)_*inter*chain_) and a decrease in the angle (*θ*) formed by the pza ligands with respect to the direction of the 1D CP chain to which they are bonded (i.e., the angle between two planes: the pz–Cd–pz plane and Cd–Cd­(N,N)–Cd plane; also, see Figure S9). (b) Structural changes observed as going from the compression side (i.e., inner arc, IA) to the expansion side (i.e., outer arc, OA) of the crystals bend: (***top***) variations in the directions along the three crystal axes, the major crystal axis, [100], and orthogonal to the bending faces, the major (01̅1)/(011̅), and minor (001)/(001̅) bending face; (***middle***) changes in the *intra*chain angles (decrease of ∠_OA_(Cl–Cd–Cl) and increase of ∠_OA_(Cd–Cl–Cd) angle) ((*i*) *x*–1, *y*, *z*; (*ii*) –*x*, 1–*y*, 1–*z*); (***bottom***) alterations in the orientation of the pza ligands in relation to the CP chain, i.e., the angle between the two planes, the pz–Cd–pz plane and Cd–Cd­(N,N)–Cd plane. (c) Comparison of the bending mechanism determined for **1** (***middle***) with mechanism of elastic bending established for [Cu­(acac)_2_] (i.e., 0D molecular building units; ***left)*** and the changes that materialize in plastic 1D crystals, containing the [Zn­(μ-X)_2_]_*n*_ backbone, when exposed to high pressure (***right***).

Furthermore, the changes in the Cd···Cd *intra*chain distances are achieved through alterations in the bridging Cd–Cl–Cd and Cl–Cd–Cl (*intra*chain) angles, the former increasing, and the latter decreasing as going from the inner to the outer side of the bend (Figure 3a,b *middle*). Consequently, the structural modulation in the polymeric spine enables the CP chains to function like large molecular springs, facilitating compression and expansion at opposite sides of the bent crystal. Thus, the structural adaptability in response to elastic bending, as long as solely CP spines are considered, aligns closely with reported elastic adaptability in a plastic crystal of 1D [M­(μ-X)_2_]_*n*_ CP under high pressure.^50^ However, further inspection of the structural details also revealed distinct differences between the two mechanisms involving 1D [M­(μ-X)_2_]_*n*_ CPs (i.e., mechanically strained elastic crystals and plastic crystals under quasi-hydrostatic pressure). In **1**, contraction of the Cd···Cd *inter*chain distance is additionally accompanied by a rotation of the pza ligands around the metal–ligand bond, resulting in a decrease in the angle (*θ*) formed by the pza plane and the plane containing Cd–Cd­(N,N’)–Cd fragments (which corresponds with the bending (011̅)/(01̅1) face; Figure 3a,b *bottom*, and S9). This is in stark contrast with the puckering of organic ligands that accompanies the spring-like changes in the CP backbone upon the increased quasi-hydrostatic pressure, and where these changes (i.e., tilting of the ligands toward the CP spine) resemble a butterfly flying motion.^48^ On the other hand, the sole ligand rotations observed in **1** are similar to rotations of the molecular building blocks (0D) in molecular crystals upon elastic bending.^24,26^

The atomic-level mechanism of elastically strained crystals of **1** thus clearly sets apart the mechanism of elasticity of 1D CPs from the two previously observed phenomena (Figure 3c). Furthermore, the changes observed in **1** also provide valuable insights into the macroscopic-level difference in elastic behavior between the two classes of compounds, namely molecular and 1D CPs crystals,^51^ with the latter generally exhibiting somewhat lower elasticity, due to accommodating the necessary changes within the 1D [M­(μ-X)_2_]_*n*_ CP spine accompanied by the rotation of the organic ligands.^52^

### Computational Approach to Structural Mobility upon Elastic Bending

To gain further insight into the atomic-level structural alterations that were not directly discernible through the structural investigations, we directed our subsequent computational studies toward simulating the tensile and compressive forces acting on the opposing sides of a bent crystal (Figure 4a,b).

**Figure 4:**
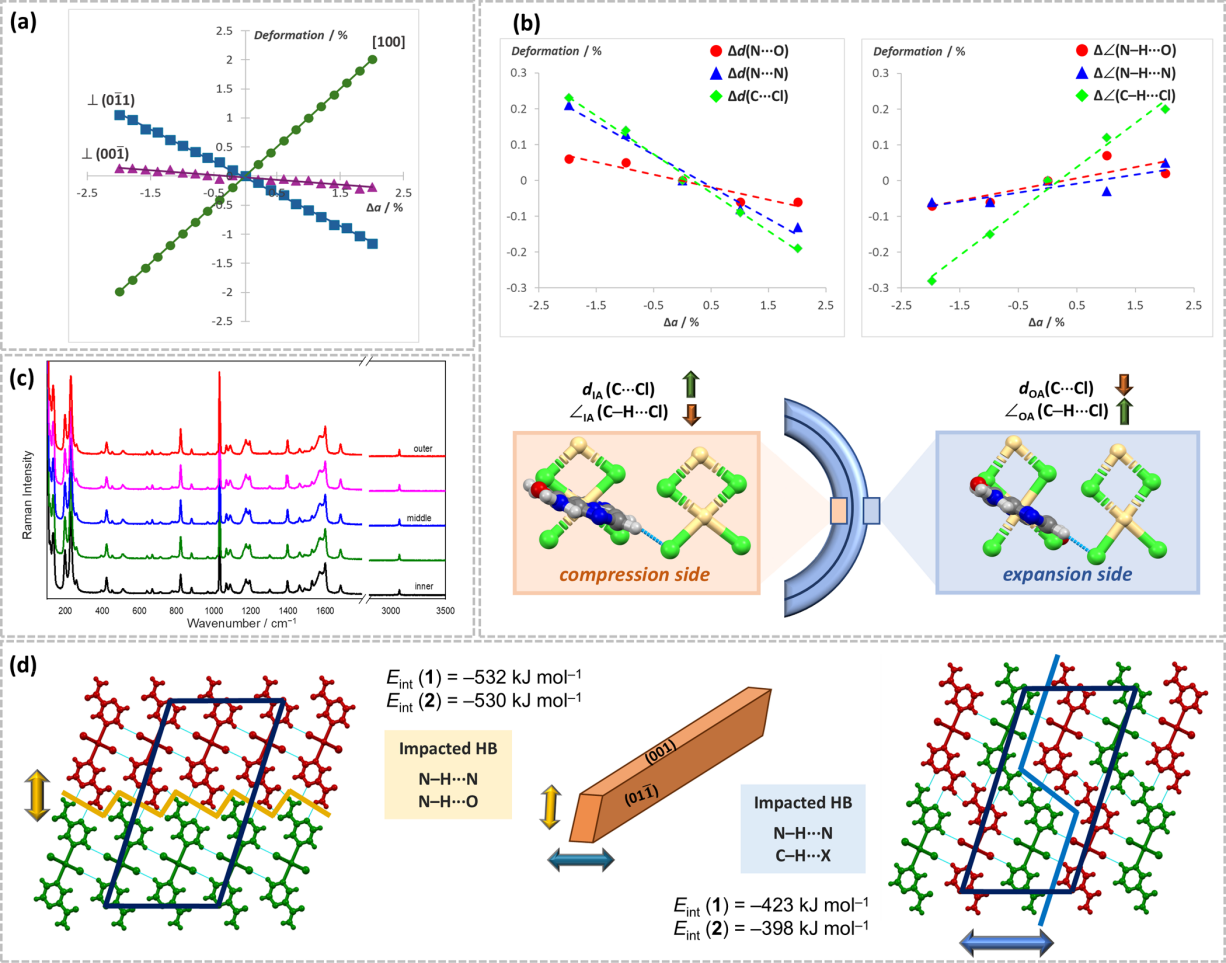
(a) The calculated deformations along the two directions associated with the bending faces in **1** (i.e., orthogonal to (001̅) and (011̅)) that materialize in response to the imposed uniaxial strain in the *a* direction within the range of –2% to +2%. (b) The visualization of calculated changes in hydrogen-bond geometry due to the imposed strain (top left: HB distances; top right: HB angles), and illustration of the corresponding structural details at the interior (IA; left) and exterior (OA; right) of the bent crystal (under imposed strains); compression of the CP backbone at the interior of the bent crystal leads to an increase in interchain distances, resulting in enlargement of the hydrogen-bonding *d*_IA_(C···Cl) distance and a deviation of the ∠_IA_(C–H···Cl) angle from linearity; conversely, opposite alterations materialize at the exterior of the bent crystal. (c) Microfocus Raman spectra recorded at five points across the crystal bend of **1**. (d) Interaction energies calculated for four neighboring 1D coordination polymers of **1** (two green- and two red-colored fragments aligned along the zigzag lines). Regions consisting of intermolecular interactions, identified as the ‘softest’ connections along the directions associated with bending faces (yellow: (001)/(001̅), left; blue: (011̅)/(01̅1), right) are highlighted by zigzag lines.

To accomplish this, we systematically subjected the unit cell to uniaxial deformation along the crystallographic *a* axis, progressing in regular increments of 0.2% within a range of −2% to +2%. Throughout the optimization procedure, we allowed the other crystallographic parameters and atomic positions to adjust, thereby ensuring the attainment of relaxed geometries.

The deformation of the *a* direction unequivocally revealed concerted yet inversely proportional changes in the other two directions, associated with the minor and major bending faces (Figure 4a), which is strongly supportive of our μ-SCXRD results. Due to this, we were confident to utilize the computational data to gain further insights into the intricate details of structural adaptability (viz. intermolecular interactions) upon elastic deformation. As a direct consequence of the increase in the *intra*chain and decrease in *inter*chain distances, at the exterior, in contrast to the interior of the bent crystal, all three hydrogen bonds become shorter and more linear as going from the convex to the concave side of the crystal (Figure 4b). However, the extent of their changes varies in both the distances and angles. The C–H···Cl, as the least influential link in the crystal structure (based on crystallographic considerations), experienced the most pronounced changes, while the opposite was found for the most influential one, the N–H···O hydrogen bond. Also, the structural mobilities during bending make a different availability of the hydrogen-bond acceptor atoms at different crystal regions, i.e., inner vs outer arc (viz., the Cl atom in the C–H···Cl in Figure 4b, *bottom*).

Our attempts to provide further experimental evidence to complement the μ-SXCRD experimental efforts and computational findings by using the μ-focus Raman technique were, unfortunately, unsuccessful. Neither shifts in vibrational bands nor clearly discernible changes in intensities were observed due to the apparent insensitivity of the method to relatively modest modifications of relevant structural features at moderate bending strains (*ε*) of **1** and **2** (Figure 4c); these were additionally confirmed by the calculated Raman spectra (for more details, see the *Microfocus Raman spectroscopy* and *Calculated Raman spectra* sections in SI).^53^

Furthermore, the trend observed from synchrotron μ-SCXRD data, evident in varying tilts of the lines indicating structural alterations along the directions orthogonal to the two different bending faces (Figure 3b, *top*), is strongly supported by computational findings (Figure 4a). They reveal even more pronounced differences in the tilts, suggesting a more significant degree of structural modifications along the directions associated with the major bending faces compared to that of the minor.

### Molecular Movements (Interaction Energies)

To elucidate the differences in tilts suggesting varying degrees of molecular movements, we calculated the interaction energies between neighboring polymeric chains in both relevant directions. Through this, we aimed to identify which direction is more susceptible to deformation.

To compute the interaction energies, we selected four neighboring 1D CPs (depicted in green and red fragments, at opposite sides of the zigzag lines; Figure 4d). We assessed the interaction energies within the regions indicated by the blue and yellow zigzag lines, which represent the regions of the weakest connectivities^54^ associated with the two directions of interest, the yellow line (N–H···N/N–H···O) influencing the deformation in the direction associated with the minor (yellow double arrow) and the blue line (N–H···N/N–H···Cl/Br) with the major bending face (blue double arrow), (for more details see SI, Figure S13).

The interactions acting across the yellow zigzag lines were significantly stronger (**1**: –532 kJ/mol; **2**: –530 kJ/mol) than those across the blue zigzag lines (**1**: –432 kJ/mol; **2**: –398 kJ/mol). This suggests less pronounced deformations along the directions associated with the minor bending faces (yellow double arrow) and a greater degree of deformation linked to the major bending face (blue double arrow); this aligns closely with our experimental and computational observations regarding different degrees of structural modification in these particular directions. Moreover, the relative difference in the intermolecular interactions is also well in line with the calculated elastic constants in these respective directions, being larger in the direction controlled by stronger and smaller in directions controlled by weaker supramolecular links. However, further considerations are required to fully differentiate the sole impact of intermolecular interactions from that of molecular geometry.

This finding offers further insight into the mechanisms that govern crystal elastic properties, which is crucial for advancing the use of these materials in applications that require controlled mechanical behavior.

## Conclusions

By investigating the elastic properties of two crystalline 1D coordination polymers, we identified the first mechanism of mechanically stimulated elasticity in crystals of 1D materials with atomic-level precision. The structural mobilities in elastically strained crystals of the 1D CP contrast with those seen in elastic molecular crystals (0D) subjected to mechanical force and those in plastic 1D CPs under quasi-hydrostatic pressure while still retaining certain similarities. The observed spring-like mobility of the polymeric spine, which expands externally and compresses internally in the bent crystal of **1**, is driven by the concerted adaptations of the Cd···Cd *intra*chain distances achieved through modifications of the bridging Cd–Cl–Cd/Cl–Cd–Cl angles, and rotations of the organic ligands. This behavior mirrors the mobility of the 1D polymeric spines under quasi-hydrostatically stimulated elasticity while simultaneously resembling ligand adjustments found in the elastically flexible molecular crystals under mechanical stress.

Moreover, by integrating experimental observations with computational insights, we have derived direct links between the relative degree of structural changes in directions associated with the bending faces with the relative strength of intermolecular connections and crystal stiffness (*E*) influencing these particular directions. The weaker the supramolecular connectivity in the direction toward the bending crystal faces, the more susceptible the crystal structure becomes to adjustments in that direction when subjected to mechanical stress.

Unveiling the mechanisms of the crystal elasticity of 1D coordination polymers, we made a firm foundation for facilitating the engineering of elastic properties of 1D crystalline materials, which is crucial for fully tapping into the transformative potential of these flexible materials in shaping the technologies of tomorrow.

## Supplementary Material











## Notes

synthetic procedures for **1** and **2**; PXRD; details of SCXRD on straight crystals; synchrotron microfocus SCXRD on bent crystals; computed elastic moduli, and interaction energies; microfocus Raman spectra; modified three-point bending experiments; AFM measurements (PDF)
Bending of 1 (force applied to major face) (Movie 1) (AVI)
Bending of 1 (force applied to minor face) (Movie 2) (AVI)
Bending of 2 (force applied to major face) (Movie 3) (AVI)
Bending of 2 (force applied to minor face) (Movie 4) (AVI)
