# Supplementary material for: Unveiling Structure-Dynamic Processes in Crystals of 1D Cd(II) Coordination Polymers during the Elastic Flexible Events
Source: J Am Chem Soc. 2025 Jun 9;147(25):22219–27. doi: 10.1021/jacs.5c07191 (PMC12203600; doi:10.1021/jacs.5c07191)
Supplement: Supplementary file 1 [file ja5c07191_si_001.pdf]

Unveiling Structure-Dynamic Processes in Crystals of 1D Cd(II) Coordination Polymers during the Elastic Flexible Events

Marijana Đaković,[a,]* *Mateja Pisačić,*[a] *Mladen Borovina,*[a] *Ivan Kodrin,*[a] *Adriana Kenđel,*[a]

Tea Frey[a]

[a] Department of Chemistry, Faculty of Science, University of Zagreb, Zagreb 10000, Croatia

Supplementary Information

Table of Contents

1. Synthetic procedure 3
2. Powder X-ray crystallography 4
3. Single crystal X-ray crystallography 5
4. Synchrotron microfocus SCXRD 8
5. Computational studies 16
6. Microfocus Raman spectroscopy 24
7. Crystal bending experiments 25
8. AFM measurements 30

1. Synthetic procedure

General considerations. Materials and measurements. All metal salts, precursors and solvents were purchased from commercial suppliers and used without further purification.

Experimental procedure for the preparation of [CdX_2(pza)$_2$]$_n$

Cadmium(II) salt (CdX_2, 1 eq.) was dissolved in water (5,0 mL) and pyrazinamide (pza, 2 eq.) in ethanol (96%, 10,0 mL). The resulting colorless solutions were mixed and stirred. In the case of both compounds (**1** and **2**), a white precipitate was immediately formed. Stirring continued for an additional 30 minutes, and the product was filtered off, washed with small amounts of cold water, and dried in air.

The powder diffraction patterns for **1** and **2** (bulk sample) were consistent with those calculated from single crystal data (Fig. S1).

[$CdCl_2$(pza)$_2$]$_n$ (**1**). Used: $CdCl_2 \cdot H_2O$ hydrate (0.10 g; ~ 0.50 mmol), pza (0.126 g; ~ 0.10 mmol). Microanalysis: Calcd. for $C_{10}H_{10}CdCl_2N_6O_2$ (M_r = 429.54): C, 27.96; H, 2.35; N, 19.57% Found: C, 27.90; H, 2.40; N, 19.62%.

[$CdBr_2$(pza]$_n$ (**2**). Used: $CdBr_2 \cdot 4H_2O$ (0.176 g, ~0.49 mmol), pza (0.123 g; ~ 0.10 mmol). Microanalysis: Calcd. for $C_{10}H_{10}CdBr_2N_6O_2$ (M_r = 518.45): C, 23.17; H, 1.95; N, 16.21% Found: C, 23.20; H, 1.90; N, 16.26%.

Growing crystals of 1 and 2 by the layering technique - general procedure

Cadmium(II) salt (CdX_2) was dissolved in water and pyrazinamide (pza) in ethanol (w = 96%). The resulting aqueous solution of the cadmium(II) salt was placed in a test tube, layered with 1 mL of ethanol (w = 96%), and then by the ethanol solution of the ligand. The test tubes were sealed using parafilm and left standing undisturbed. After a week a few holes in the parafilm were formed to allow slow evaporation. Colorless needle-like crystals of **1** and **2** were harvested in approx. two weeks.

[$CdCl_2$(pza)$_2$]$_n$ (**1**). Used: $CdCl_2$ aqueous solution (4.5 mL, 0.11 mol dm^{-3}), pza ethanol solution (9 mL, 0.10 mol dm^{-3}).

[$CdBr_2$(pza)$_2$]$_n$ (**2**). Used: $CdBr_2$ aqueous solution (2.0 mL, 0.05 mol dm^{-3}), pza ethanol solution (4.0 mL, 0.05 mol dm^{-3}).

2. Powder X-ray crystallography

X-ray powder diffraction experiments were performed on a Philips PW 1850 diffractometer, CuKα radiation, 40 kV voltage, and 40 mA current. The patterns were collected in the angle region between 5° and 50° (2ϑ) with a step size of 0.02°.

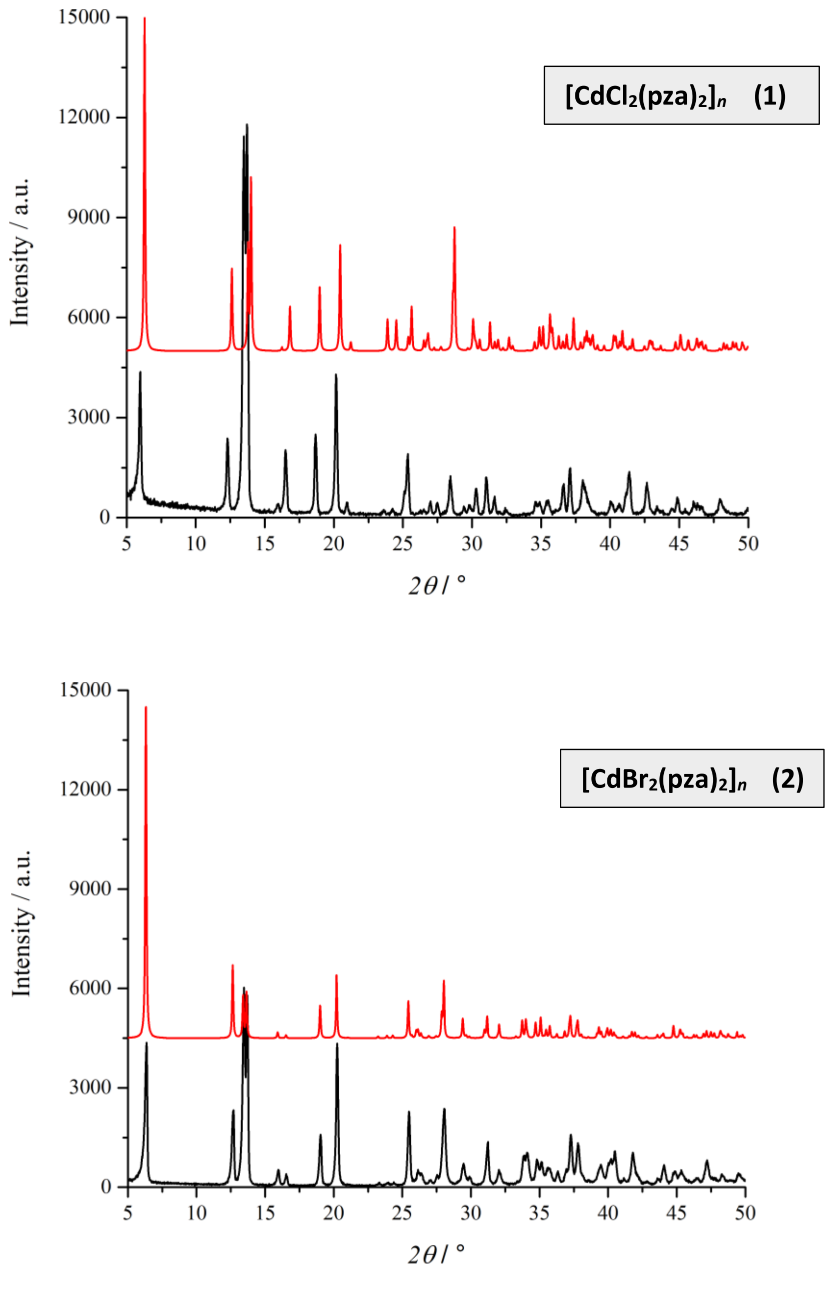

Figure S1. Overlay of PXRD traces for **1** and **2** (experimental, **black**, and calculated, **red**).

3. Single crystal X-ray crystallography

Data collection on straight crystals. Single crystals **1** and **2** were mounted on a glass fiber and were fixed with super glue. Data collection was carried out at room temperature, 295(2) K, on an XtaLAB Synergy-S Dualflex diffractometer equipped with a PhotonJet (Mo) microfocus X-ray source and a HyPix-6000HE hybrid photon counting (HPC) X-ray area detector. Data collection and reduction, including absorption correction, was performed using CrysAlisPro software (version 1.171.41.93a).[i] The structures were determined using the Olex2[ii] interface (version 2.13). The starting structural model was obtained using SHELXT[iii] and refined on F^2 with the SHELXL[iv] algorithm. Thermal ellipsoids were drawn at the 50% probability level with Mercury, Version 2024.1.0.[v] CCDC Nos. 2371911 and 2371913 contain supplementary crystallographic data.
Data can be obtained free of charge from the Cambridge Crystallographic Data Centre via https://www.ccdc.cam.ac.uk/structures/.

Table S1. Crystallographic and structure refinement parameters for **1** and **2**.

**Compound**	**$[CdCl_2(pza)_2]_n$ (1)**	**$[CdBr_2(pza)_2]_n$ (2)**
Formula	$C_{10}H_{10}CdCl_2N_6O_2$	$C_{10}H_{10}CdBr_2N_6O_2$
M_r	429.54	518.46
**Crystal**	**Undeformed**	**Undeformed**
Colour and habit	Colorless needle, straight	Colorless needle, straight
Crystal dimensions (mm^3)	0.19 × 0.03 × 0.01	0.24 × 0.02 × 0.02
Crystal system	Triclinic	Triclinic
Space group	$P\bar{1}$	$P\bar{1}$
a (Å)	3.73890(10)	3.84270(10)
b (Å)	6.5674(2)	6.7617(2)
c (Å)	14.2918(4)	14.2869(3)
α (°)	100.989(3)	101.253(2)
β (°)	90.852(2)	90.883(2)
γ (°)	95.102(2)	95.502(2)
V (Å^3)	342.952(17)	362.184(16)
Z	1	1
Temperature (K)	295(2)	295(2)
D_{calc} (g cm^{-3})	2.080	2.377
Radiation wavelength λ (Å), μ (mm^{-1})	0.71073, 1.994	0.71073, 7.036
F (000)	210	246
Scan type	ω	ω
θ range for data collection (°)	4.36 – 29.99	2.91 – 30.5
h, k, l range	–5:5, –9:9, –20:20	–5:5, –9:9, –20:20
No. measured reflections	15813	22094
No. Independent reflections	1998	2222
No. Observed reflections, $I \geq 2\sigma$	1908	1804
Completeness	99.8%	99.9%
No. refined parameters	105	97
R, wR [$I \geq 2\sigma$]	0.0244, 0.0552	0.0265, 0.0586
R, wR [all data]	0.0264, 0.0559	0.0371, 0.0611
Goodness of fit on F^2, S	1.078	1.056
Max, min. El. density (e Å^{-3})	–0.599, 0.452	–0.593, 0.601
CCDC number	2371911	2371913

Table S2. Selected bond lengths (Å) and angles (°) for **1** and **2**.

**Compound**	**$[CdCl_2(pza)_2]_n$**	**$[CdBr_2(pza)_2]_n$**
**Crystal**	**Undeformed**	
Bonds		
Cd—X1	2.6000(4)	2.7196(3)
Cd—X1*i*	2.6025(5)	2.7156(3)
Cd—N1	2.4154(17)	2.449(2)
O1—C5	1.229(3)	1.234(4)
N1—C1	1.338(2)	1.332(3)
N1—C4	1.337(3)	1.339(3)
N2—C2	1.336(3)	1.340(4)
N2—C3	1.341(3)	1.331(4)
N3—C5	1.317(3)	1.318(4)
C1—C2	1.378(3)	1.386(4)
C2—C5	1.511(3)	1.500(4)
C3—C4	1.386(3)	1.388(4)
Angles		
Cd1—X1—Cd1*ii*	91.889(15)	89.985(8)
X1—Cd1—X1*i*	91.889(15)	89.985(9)
X1—Cd1—X1*iii*	88.111(15)	90.015(9)
N1—Cd1—X1	90.04(4)	90.11(5)
N1—Cd1—X1*i*	88.01(4)	87.65(6)
N1—Cd1—X1*iii*	91.99(4)	92.35(6)
N1—Cd1—X1*iv*	89.96(4)	89.89(5)

X = Cl for **1** and Br for **2**
Symmetry operators: (*i*) *x* + 1, *y*, *z*; (*ii*) *x* – 1, *y*, *z*; (*iii*) – *x*, 1 – *y*, 1 – *z*; (*iv*) 1 – *x*, 1 – *y*, 1 – *z*.

Table S3. Hydrogen bond distances (Å) and angles (˚) for **1** and **2** (undeformed crystals).

D–H···A	*d*(H···A)/Å	*d*(D···A)/Å	∠ (D–H···A)/˚	R_{HA}	R_{DA}
**1**					
C4–H4···Cl1*i*	2.89	3.749(2)	153.8	0.98	1.09
N3–H3B···N2*ii*	2.61(2)	3.236(3)	133(2)	0.95	1.04
N3–H3A···O1*iii*	2.00(2)	2.864(2)	171(3)	0.74	0.93
**2**					
C4–H4···Br1*i*	3.01	3.861(3)	152.9	0.98	1.08
N3–H3B···N2*ii*	2.66(4)	3.280(3)	131(4)	0.97	1.06
N3–H3A···O1*iii*	2.04(2)	2.882(4)	173(4)	0.75	0.94

Symmetry codes: (*i*) *x* + 1, *y* – 1, *z*; (*ii*) – *x* + 1, – *y*, – *z*; (*iii*) – *x*, – *y* + 1, – *z*.
[a] The normalized distance, *R*, is defined according to Lommerse *et al.*[vi] $R_{HA} = d(H\cdots A) / (r_H + r_A)$; $R_{DA} = d(D\cdots A) / (r_D + r_A)$ where r_H, r_D and r_A are the Bondi van der Waals radii of the hydrogen atom and respective hydrogen-bond donor and acceptor atoms (H 1.20 Å, Cl 1.75 Å, Br 1.86 Å, O 1.52 Å, N 1.55 Å, C 1.70 Å) in the D—H···A hydrogen bond.

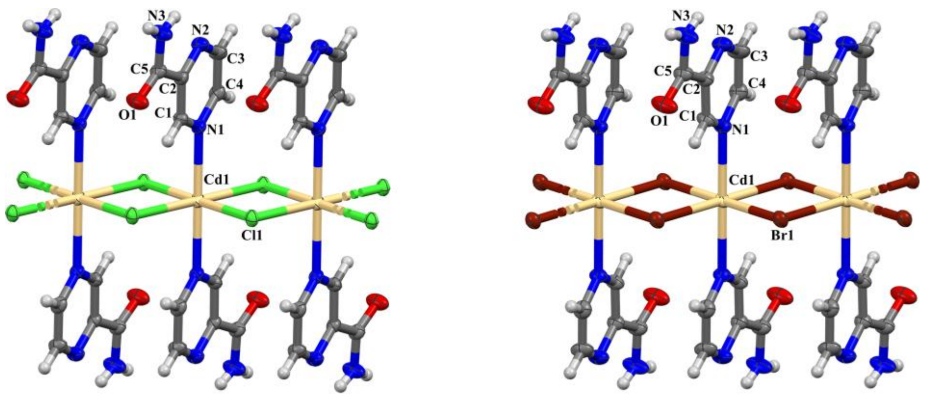

(a) (b)

Figure S2. ORTEP-style plot of **1** and **2** with labeling scheme of the asymmetric unit. Thermal ellipsoids are drawn at 50% probability level at 295(2) K. The Cd···Cd intrachain distance corresponds to the unit cell parameter *a*.

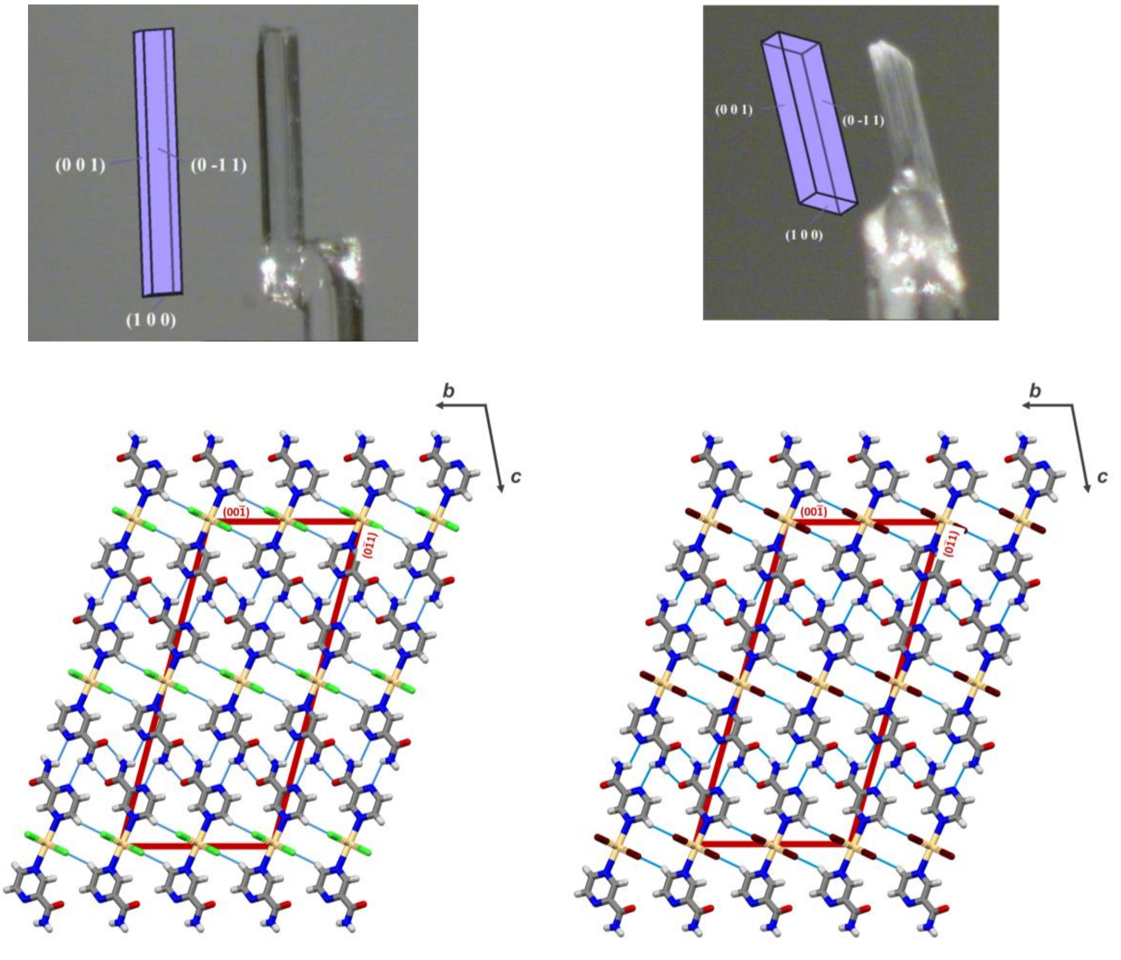

(a) (b)

Figure S3. Crystal morphology with the face indices and crystal packing of **1** (a) and **2** (b) viewed down the *a* axis. Crystal faces (001)/($00\bar{1}$)/($01\bar{1}$)/($0\bar{1}1$) are indicated as red lines. The adjacent polymeric chains are linked via self-complementary N–H···O, N–H···N and C–H···Cl/Br hydrogen bonds (shown as blue dotted lines).

4. Synchrotron microfocus SCXRD

Crossed polarized light was used to identify single crystals of good quality using a Zeiss Stemi 508 Microscope in transmission mode. Crystals **1** and **2** were glued in a bent form on a magnetic base holder and placed on a goniometer head. The crystals were oriented so that the beam's trajectory was perpendicular to the loop of the crystal.

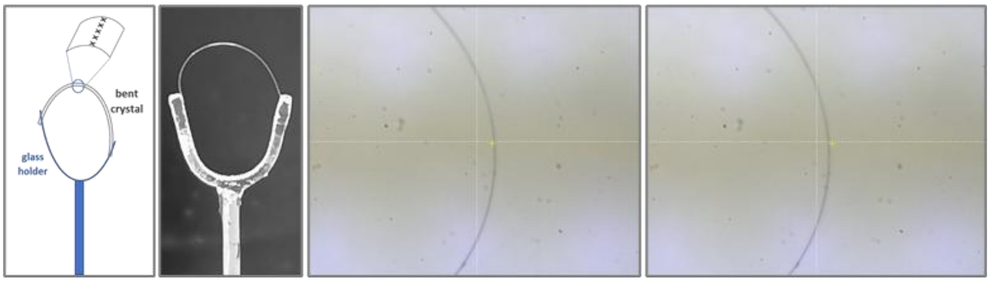

Figure S4. Schematic and photograph representing a crystal in a bent form and a mapping experiment using a microfocus X-ray beam.

General. Data were collected on the X06SA (PXI) beamline at the Swiss Light Source (Paul Scherrer Institute, Switzerland) equipped with an LN2-cooled fixed-exit Si(111) monochromator and EIGER 16M X detector. All measurements were performed at room temperature using a wavelength λ = 0.72932 Å and a microfocused 5 µm × 7.5 µm X-ray beam (full width at half-maximum) with the *DA+* data acquisition software.[vii]

Data collections for structure determination (at the inner and outer arcs of bent crystals) of* 1 *and* 2*. A point at the maximal curvature of the bent crystal was selected, and the crystal was positioned so that only a small portion of the outer (OA) or inner arc (IA) of the crystal was in the beam, and at each point (OA and IA) a φ scan was performed; additional experimental details concerning data collection are listed in Table S4.

The structures were determined using the Olex2[ii] interface (version 2.13). The starting structural model was obtained using SHELXT[iii] (**1**) or SHELXS[iv] (**2**) and refined on F^2 with the SHELXL[iv] algorithm. Anisotropic displacement parameters were used to model heavier atoms (Cd and Cl/Br), while isotropic displacement parameters were used to model remaining non-hydrogen atoms (C, N, O). In the structure of the inner arc (IA) region of **1**, the bond lengths in the pyrazinamide ligands (pza) were restrained using dfix commands, while corresponding atom positions in all other instances were refined freely. Hydrogen atoms (both aromatic and amide) were generated geometrically using the riding model with the isotropic factor set at 1.2 U_{eq}. Due to the applied restraints, crystallographic data were not used to understand the difference in HB geometries at the two arcs of the bent (IA, OA).

***Data collections for mapping experiments of* 1.** Data collection for the mapping experiment was performed exclusively for **1**, as the bent crystals of **2** suffered significant radiation damage, which hindered their ability to withstand the data collection process.

A linear scan of the bent crystal of **1** was conducted starting at the outmost point towards the innermost point at regular increments of 2 µm. At each point, a φ scan was performed, and 150 diffraction images were collected with an oscillation angle of 0.2° (total width of 30°) and an exposure time of 1.0 s. Data integration and reduction were performed using CrysAlisPro (version 1.171.41.93a).

The structures were determined using the Olex2[ii] interface (version 2.13). The starting structural model was obtained using the crystal structure determined at the inner arc (IA) of the bent crystal of **1** and refined on F^2 with the SHELXL[iv] algorithm.

CCDC Nos. 2371910, 2371912, 2371914 and 2371915 (for structure determination) and CCDC Nos. 2448975–2448982 (for mapping experiments), contain supplementary crystallographic data. Data can be obtained free of charge from the Cambridge Crystallographic Data Centre via https://www.ccdc.cam.ac.uk/structures/.

Table S4. Synchrotron μ-SCXRD crystallographic and structure refinement parameters for **1** and **2**.

	**$[CdCl_2(pza)_2]_n$**		**$[CdBr_2(pza)_2]_n$**	
Formula<br>M_r	$C_{10}H_{10}CdCl_2N_6O_2$<br>429.54		$C_{10}H_{10}CdBr_2N_6O_2$<br>518.46	
**Crystal**	**Bent, inner arc (IA)**	**Bent, outer arc (OA)**	**Bent, inner arc (IA)**	**Bent, outer arc (OA)**
Color and habit	Colorless needle, bent	Colorless needle, bent	Colorless needle, bent	Colorless needle, bent
Crystal dimensions (mm^3)	0.04 × 0.02	0.04 × 0.02	0.05 × 0.03	0.05 × 0.03
Crystal system	Triclinic	Triclinic	Triclinic	Triclinic
Space group	$P\bar{1}$	$P\bar{1}$	$P\bar{1}$	$P\bar{1}$
a (Å)	3.7235(8)	3.7521(3)	3.8379(4)	3.8558(2)
b (Å)	6.5739(18)	6.5541(8)	6.7751(10)	6.7675(4)
c (Å)	14.291(2)	14.2712(10)	14.2549(14)	14.2684(7)
α (°)	100.88(2)	100.832(9)	101.248(10)	101.154(4)
β (°)	90.84(2)	90.811(7)	90.802(8)	90.796(4)
γ (°)	94.95(2)	95.129(8)	95.415(9)	95.441(4)
V ($Å^3$)	342.07(13)	343.15(6)	361.71(7)	363.44(3)
Z	1	1	1	1
Temperature (K)	295(2)	295(2)	295(2)	295(2)
D_{calc} (g cm^{-3})	2.085	2.079	2.380	2.369
Radiation wavelength λ (Å), μ (mm^{-1})	0.72932, 2.130	0.72932, 2.124	0.72932, 7.514	0.72932, 7.478
#frames collected, φ angle, scan width	150, 30°, 0.2°	200, 40°, 0.2°	200, 40°, 0.2°	200, 40°, 0.20°
F (000)	210	210	246	246
Scan type	φ	φ	φ	φ
θ range for data collection (°)	5.64 – 27.46	5.75 – 28.92	6.05 – 28.78	5.45 – 28.84
h, *k*, *l* range	–4:4, –6:7, –11:0	–4:4, –6:7, –13:1	–4:4, –6:7, –2:15	–4:4, –7:6, –14:1
No. measured reflections	360	524	564	581
No. Independent reflections	355	509	543	561
No. Observed reflections, $I \geq 2\sigma$	322	500	507	521
Completeness	24.6%	30.5%	31.3%	29.9%
No. refined parameters	52	52	52	52
R, *wR* [$I \geq 2\sigma$]	0.0750, 0.1992	0.0616, 0.1646	0.0585, 0.1733	0.0470, 0.1341
R, *wR* [all data]	0.0802, 0.2094	0.0624, 0.1663	0.0596, 0.1751	0.0482, 0.1352
Goodness of fit on F^2, *S*	1.066	1.105	1.206	1.182
Max, min. El. density (e $Å^{-3}$)	–0.766, 0.820	–0.825, 1.041	–0.677, 0.730	–0.592, 0.551
CCDC number	2371914	2371915	2371910	2371912

Table S5. Synchrotron μ-SCXRD data: selected bond lengths (Å) and angles (°) for **1** and **2**.

	**$[CdCl_2(pza)_2]_n$**		**$[CdBr_2(pza)_2]_n$**	
	**Bent, IA**	**Bent, OA**	**Bent, IA**	**Bent, OA**
Bonds				
Cd—X1	2.600(5)	2.598(3)	2.7203(10)	2.7226(8)
Cd—X1^{i}	2.602(5)	2.607(3)	2.7148(11)	2.7195(10)
Cd—N1	2.44(2)	2.428(15)	2.461(15)	2.442(13)
O1—C5	1.231(17)	1.247(18)	1.233(19)	1.241(15)
N1—C1	1.336(19)	1.30(2)	1.36(2)	1.33(2)
N1—C4	1.360(17)	1.353(17)	1.329(16)	1.342(14)
N2—C2	1.332(17)	1.350(18)	1.336(18)	1.346(15)
N2—C3	1.358(19)	1.32(2)	1.32(3)	1.32(2)
N3—C5	1.32(2)	1.32(3)	1.31(3)	1.32(3)
C1—C2	1.364(19)	1.39(3)	1.37(3)	1.36(2)
C2—C5	1.485(18)	1.47(2)	1.47(2)	1.46(2)
C3—C4	1.38(2)	1.39(3)	1.37(3)	1.36(3)
Angles				
Cd1—X1—Cd1^{ii}	91.4(2)	92.24(11)	89.84(4)	90.23(3)
X1—Cd1—X1^{i}	91.4(2)	92.24(11)	89.84(4)	90.23(3)
X1—Cd1—X1^{iii}	88.6(2)	87.76(11)	90.16(4)	89.77(3)
N1—Cd1—X1	89.5(4)	89.7(3)	90.4(2)	90.1(2)
N1—Cd1—X1^{i}	88.0(4)	87.5(3)	87.9(2)	87.7(2)
N1—Cd1—X1^{iii}	92.0(4)	92.5(3)	92.1(2)	92.3(2)
N1—Cd1—X1^{iv}	90.5(4)	90.3(3)	89.6(2)	89.9(2)

X = Cl for **1** and Br for **2**

Symmetry operators: (*i*) *x* + 1, *y*, *z*; (*ii*) *x* – 1, *y*, *z*; (*iii*) – *x*, 1 – *y*, 1 – *z*; (*iv*) 1 – *x*, 1 – *y*, 1 – *z*.

Table S6. Comparison of the Cd···Cd distances of the straight crystal and the inner and outer sections of the bent crystals for **1** and **2**.

Compound		Cd1···Cd1^{i} (Å)
**1**	Inner arc	3.724(1)
	Straight crystal	3.7389(1)
	Outer arc	3.752(1)
**2**	Inner arc	3.838(1)
	Straight crystal	3.8427(1)
	Outer arc	3.856(1)

Symmetry code: (*i*) –1 + *x*, *y*, *z*.

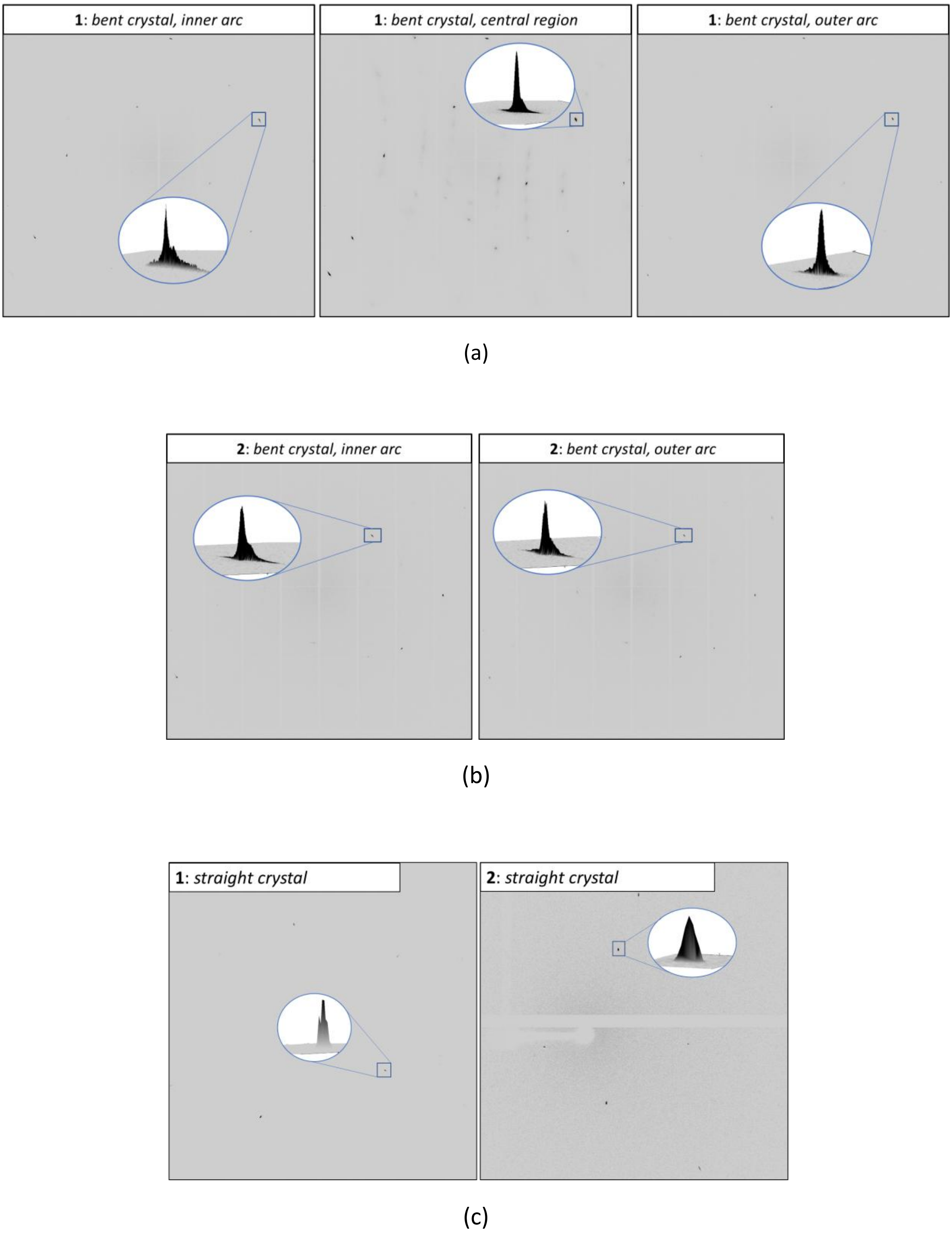

Figure S5. Diffraction images and peaks profiles for **1** and **2**; (a) at the inner arc, central region and outer arc of the bent crystal of **1**, (b) inner and outer arcs of the bent crystal of **2**, and (c) on the straight crystals of **1** and **2**.

Table S7. Synchrotron μ-SCXRD data: Selected crystallographic data, bond distances and angles collected at different spots on the arc of the bent crystal of **1** with a synchrotron microfocus X-ray beam. Point P1 corresponds to the inner arc (IA) region, and P8 corresponds to the outer arc (OA) region of the bent crystal.

**$[CdCl_2(pza)_2]_n$**								
Spot	P1	P2	P3	P4	P5	P6	P7	P8
Relative position	–7 μm	–5 μm	–3 μm	–1 μm	1 μm	3 μm	5 μm	7 μm
Crystallographic data								
a (Å)	3.7282(2)	3.7293(3)	3.7320(3)	3.7351(3)	3.7387(3)	3.7436(3)	3.7471(3)	3.7499(3)
b (Å)	6.5931(8)	6.5891(9)	6.5851(9)	6.5755(9)	6.5676(9)	6.5642(9)	6.5633(9)	6.5627(8)
c (Å)	14.2999(9)	14.2934(9)	14.2902(9)	14.2793(9)	14.2723(8)	14.2720(9)	14.2694(8)	14.2624(8)
α (°)	100.730(10)	100.750(11)	100.752(11)	100.758(11)	100.710(10)	100.726(11)	100.797(10)	100.764(9)
β (°)	90.900(6)	90.924(7)	90.875(7)	90.877(8)	90.888(8)	90.850(8)	90.831(7)	90.862(7)
γ (°)	94.833(8)	94.858(9)	94.898(9)	94.957(9)	95.020(9)	95.039(9)	95.045(8)	95.027(8)
V (Å^3)	343.95(5)	343.64(6)	343.59(6)	343.08(6)	342.84(6)	343.08(6)	343.22(6)	343.32(5)
R, wR [all data]	0.0475, 0.1186	0.0443, 0.1009	0.0427, 0.1003	0.0412, 0.1031	0.0414, 0.1023	0.0433, 0.1079	0.0472, 0.1203	0.0473, 0.1220
Completeness	21.8%	21.4%	21.4%	21.2%	21.4%	21.3%	21.4%	21.7%
The goodness of fit on F^2, S	1.085	1.060	1.069	1.058	1.085	1.065	1.046	1.049
Max, min. el. density (e Å^{-3})	–0.496, 0.429	–0.446, 0.417	–0.442, 0.449	–0.413, 0.414	–0.430, 0.399	–0.429, 0.423	–0.403, 0.468	–0.401, 0.469
CCDC number	2448975	2448976	2448977	2448978	2448979	2448980	2448981	2448982
Bond distances								
Cd—Cl1	2.595(3)	2.594(2)	2.596(2)	2.597(2)	2.596(2)	2.596(2)	2.597(2)	2.596(2)
Cd—Cl1^{i}	2.599(3)	2.601(3)	2.601(3)	2.603(2)	2.603(2)	2.602(3)	2.604(3)	2.604(3)
Cd—N1	2.422(16)	2.426(14)	2.429(14)	2.433(13)	2.428(13)	2.423(13)	2.424(14)	2.432(15)
Bond angles								
Cd1—Cl1—Cd1^{ii}	91.75(11)	91.76(10)	91.81(10)	91.83(9)	91.98(9)	92.13(10)	92.19(10)	92.28(10)
Cl1—Cd1—Cl1^{i}	91.75(11)	91.76(10)	91.81(10)	91.83(9)	91.98(9)	92.13(10)	92.20(10)	92.28(10)
Cl1—Cd1—Cl1^{iii}	88.25(11)	88.24(10)	88.19(10)	88.17(9)	88.02(9)	87.87(10)	87.80(10)	87.72(10)

Symmetry operators: (*i*) $x + 1, y, z$; (*ii*) $x – 1, y, z$; (*iii*) $– x, 1 – y, 1 – z$.

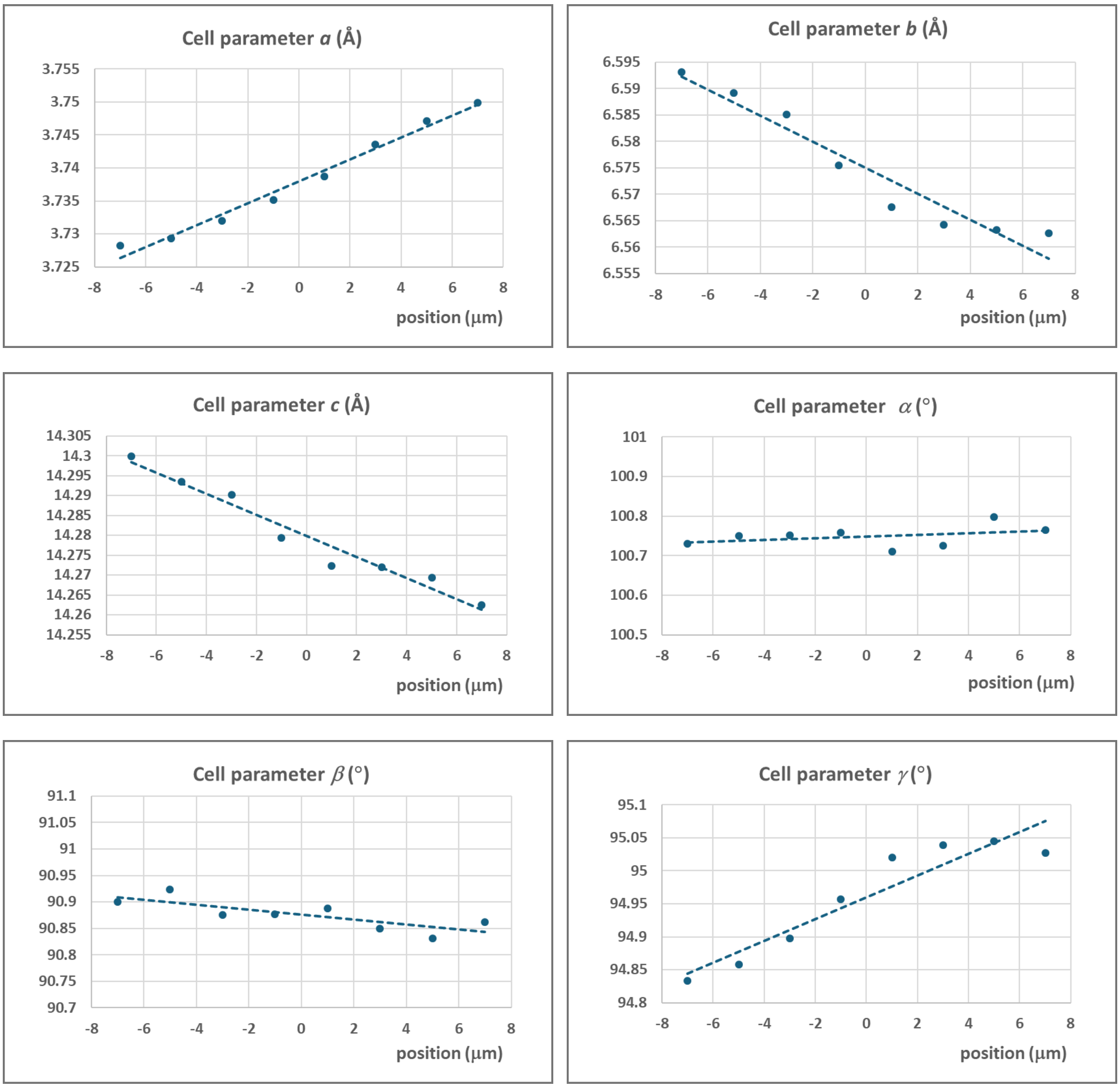

Figure S6. Changes in the unit cell parameters from the concave to the convex side of the bent crystal of **1**. The unit cell parameter *a* corresponds to the intrachain Cd···Cd distance.

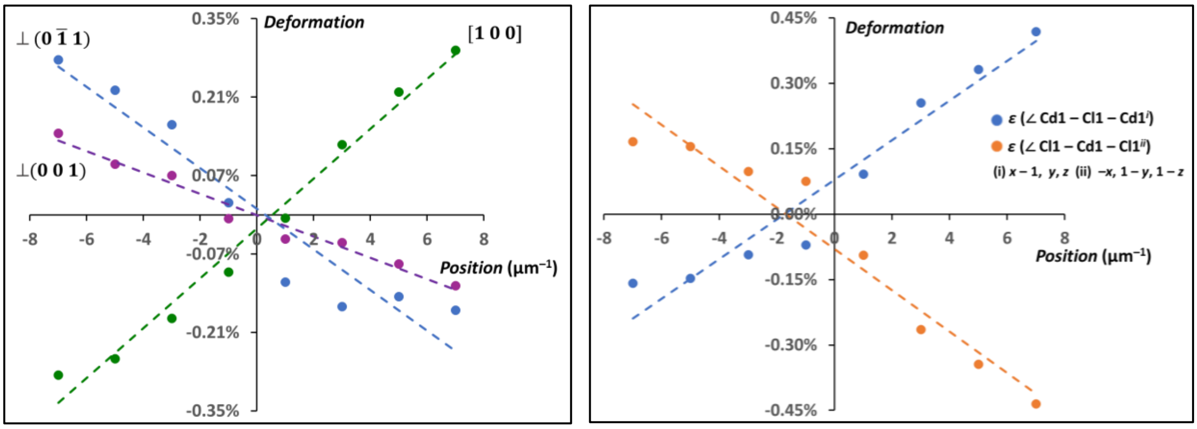

Figure S7. ***Left:*** Deformation (ε) along the directions perpendicular to the bending faces (001)/($00\bar{1}$) and ($01\bar{1}$)/($0\bar{1}1$) and along the *a* axis from the inner arc (left) to the outer arc (right) of the bent crystal of $[CdCl_2(pza)_2]_n$ (**1**); presenting the deformation orthogonally to the bending faces delivered precise enough representation of the structural alterations, while effectively accounting for the changes in crystal morphology induced by the bending of the triclinic system. ***Right:*** Deformation (ε) of ∠(Cd1–Cl1–Cd1) and ∠(Cl1–Cd1–Cl1) angles between cadmium and chloride atoms in the polymeric chain from the inner arc (left) to the outer arc (right) of the bent crystal of $[CdCl_2(pza)_2]_n$ (**1**).

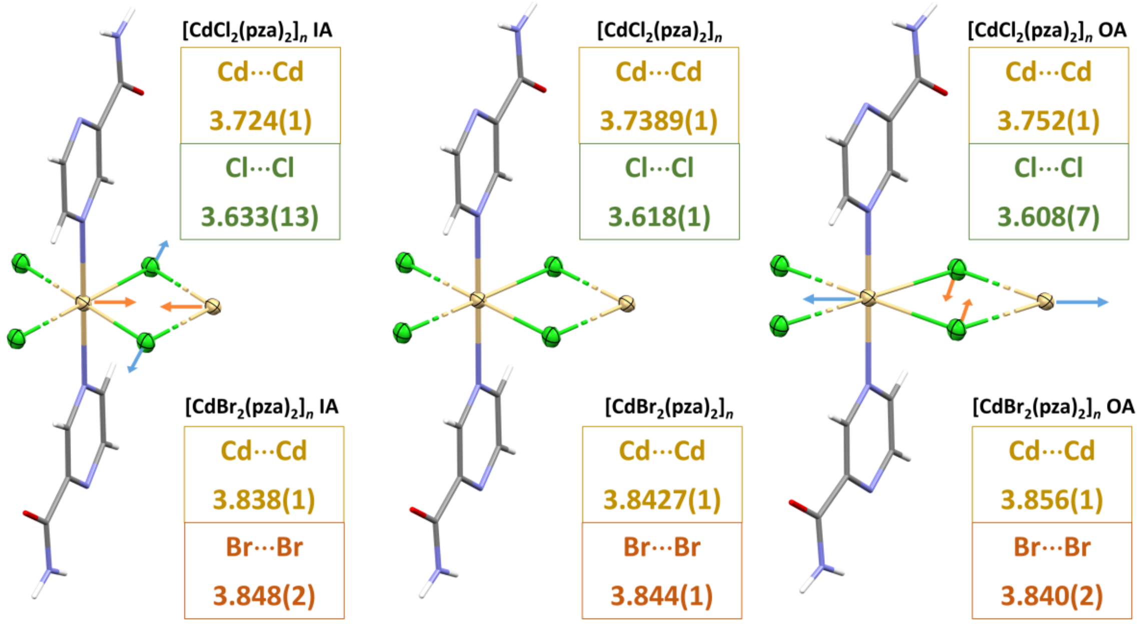

Figure S8. Structural changes observed in the polymeric spine in the inner arc (left), unbent crystal (middle), and outer (right) arc of a bent crystal of $[CdCl_2(pza)_2]_n$ (**1**). The depiction of atomic displacement is enhanced for illustrative clarity. Compression of the polymeric chain is observed in the inner arc region, seen as a decrease in the Cd···Cd distances (i.e. decrease of the unit cell parameter *a*) coupled with the in-plane displacement of the halogen atoms, which results in the enlarged X–Cd–X angles and decreased Cd–X–Cd angles. The opposite trend is observed in the outer arc region where the polymeric chain expands, leading to an increase in the Cd···Cd distances (i.e. increase of the unit cell parameter *a*) coupled with the in-plane displacement of the halogen atoms, which result in the smaller X–Cd–X and larger Cd–X–Cd angles.

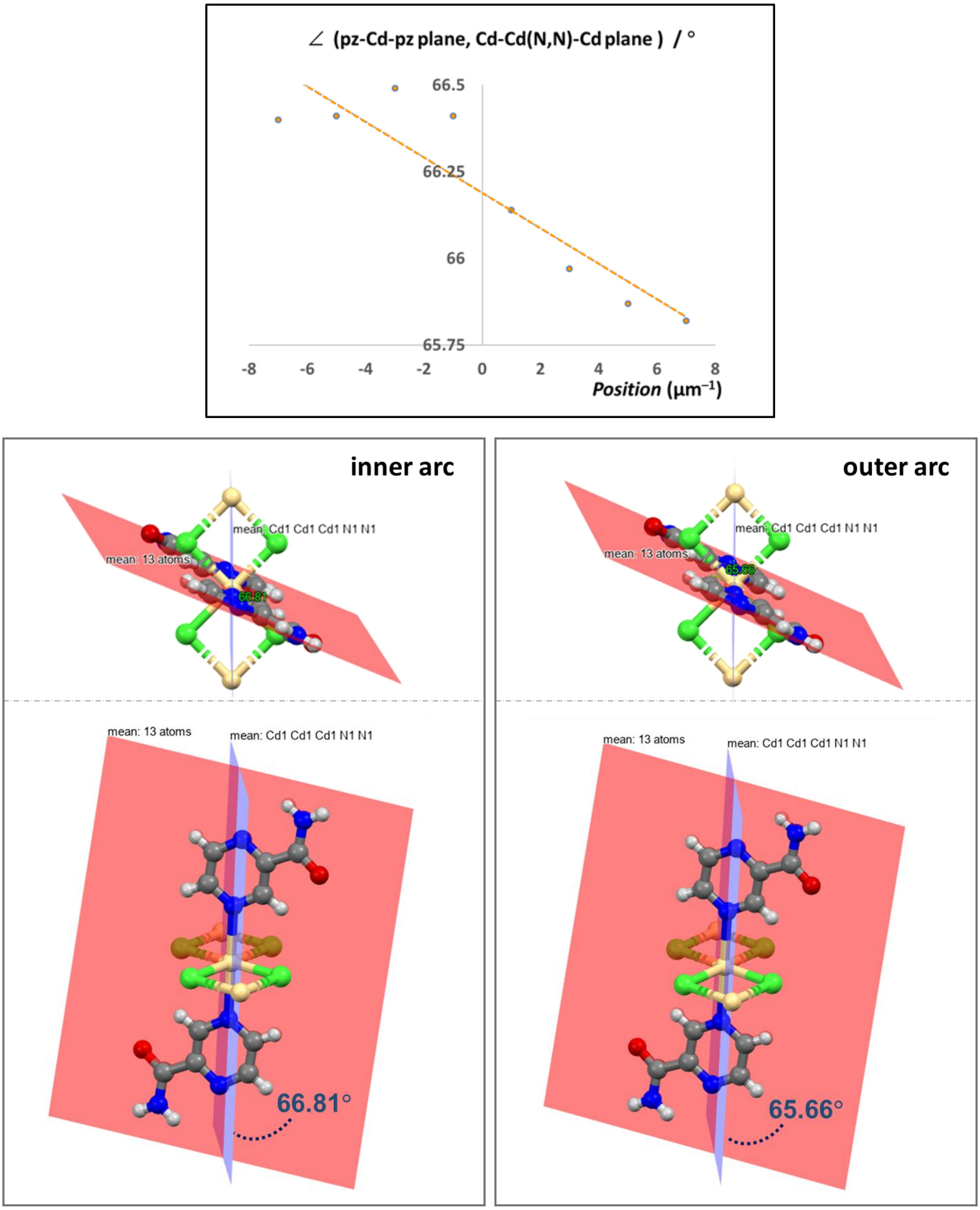

Figure S9. Changes in the angle between the planes of the pyrazinamide ligands (13 atoms: pz–Cd–pz; plane indicated in red) and the CP backbone (Cd–Cd(N_{Cd},N'_{Cd})–Cd; plane indicated in pink) as going from the inner to the outer region of the bent crystal.

5. Computational studies

Periodic density functional theory (DFT) calculations were performed in CRYSTAL17.[viii] PBE functional[ix] and Grimme's D3 correction[x] were used for a better description of weak dispersive interactions. Triple-zeta basis set pob-TZVP-rev2, adapted for periodic calculations, was employed for all atoms.[xi] Full optimization was performed with default convergence criteria. Total energy convergence was set to $10^{-7,}$ and truncation criteria for the calculations of Coulomb and exchange integrals increased to (8 8 8 8 16) for SCF calculations. Molecules were visualized in VESTA.[xii] Interaction energies E_{int} were calculated according to the formula $E_{\text{A}\cdots\text{B}} - (E_{\text{A}} + E_{\text{B}})$, where $E_{\text{A}\cdots\text{B}}$ is the energy of a fully optimized unit cell with both fragments A and B, E_{A} and E_{B} are basis set superposition error (BSSE) corrected single point energies of individual fragments A and B with the same geometry as in the optimized unit cell.[xiii] Elastic constants were calculated with a fully-automated procedure for calculating the second-order elastic constants under the default convergence criteria activated by the keyword ELASTCON in CRYSTAL17. The elastic properties were calculated from the 6 × 6 stiffness matrix and visualized in VELAS.[xiv] The crystal structure virtual tensile test was conducted on the multiple geometries derived from the initially optimized equilibrium geometry by incrementally adjusting the main crystallographic *a*-axis from −2% to +2%. In this way, the deformation of the crystal unit cell was simulated at the inner and outer arcs of the bent crystal. Options CRYDEF and FIXDEF were applied during optimization of atomic coordinates and unit cell parameters while keeping only the length of the *a*-axis constrained. Structural parameters were plotted as a function of *a*-axis stretching per cent and further analyzed at −2%, 0%, and +2%. Harmonic vibration frequencies were calculated in CRYSTAL17, applying the default convergence criteria. The Raman intensities were calculated using the Coupled-Perturbed Hartree–Fock/Kohn–Sham approach for a complete analytical calculation of Born charges. The RAMANEXP keyword was used to better match the experimental spectra. The temperature was set to 298 K, and the frequency of the incoming laser was 785 nm.

5.1. Computed elastic moduli (*E*)

To rationalize the elastic changes that the crystals undergo upon applying the external force, we computed the elastic constants using the stress-strain methodology. In principle, the total energy of a crystal can be obtained by expressing the total energy of a crystal in terms of a power series of the strain ε applied to the unit cell around the equilibrium geometry (E_0 and V_0), and σ is the corresponding stress of the unit cell:

$$E(V,\varepsilon) = E_0 + V\sum_i \sigma_i\varepsilon_i + \frac{V}{2}\sum_{ij} C_{ij}\varepsilon_i\varepsilon_j + \ldots$$

The linear term vanishes because the crystal is stress-free in the equilibrium. The elastic constants are then derived from the second derivatives of the energy to the applied strain:

$$C_{ij} = \frac{1}{V}\frac{\partial^2 E}{\partial\varepsilon_i\partial\varepsilon_j}$$

These elements of the elastic tensor for a 3D system are written as a 6 × 6 matrix. Common elastic properties can be calculated and extended from this matrix for any general direction within the crystal using the transformed compliance matrix. The following convention was adopted to align the crystal lattice with the Cartesian reference frame: *c* is parallel to *z*; *b* lies in the *y*-*z* plane at angle α to *c*; *a* is directed at angle β to *c* and angle γ to *b*.

6 × 6 matrix symmetric matrix of elastic constants (in GPa) for **1**.

29.126	15.346	13.146	−4.980	0.503	−1.974
15.346	36.522	22.732	0.779	1.249	−8.728
13.146	22.732	67.813	4.024	10.362	−5.015
−4.980	0.779	4.024	18.887	−5.762	2.180
0.503	1.249	10.362	−5.762	7.269	−3.283
−1.974	−8.728	−5.015	2.180	−3.283	9.440

6 × 6 matrix symmetric matrix of elastic constants (in GPa) for **2**.

26.482	13.050	11.963	−5.397	0.713	−1.936
13.050	32.534	19.449	1.035	1.309	−8.225
11.963	19.449	63.029	3.301	10.843	−3.666
−5.397	1.035	3.301	19.185	−6.451	3.070
0.713	1.309	10.843	−6.451	7.547	−3.512
−1.936	−8.225	−3.666	3.070	−3.512	8.217

Table S8. Calculated values of bulk modulus (in GPa), shear modulus (in GPa), Young modulus (in GPa), Poisson ratio, Ranganathan's universal anisotropy index[xv] and anisotropy of Young's modulus[xiv] for **1** and **2**.

Compound	Bulk modulus (*B*)	Shear modulus (*G*)	Young modulus (*E*)*	Poisson ratio (ν)	Ranganathan's universal anisotropy index (E_{anis})**	Anisotropy of Young's modulus (E_{anis})**
**1**	23.31	8.88	23.63	0.331	7.5	0.6
**2**	20.57	8.06	21.40	0.327	10.6	0.7

* Youngs modulus (*E*) corresponds to the Hill method (i.e. averages the outputs of the Reuss and Voigt methods for calculating elastic moduli.

** The anisotropy of the elastic moduli presented by Ranganathan's universal anisotropy index (calculated from the Voigt and Reuss bulk and sheer moduli) and Anisotropy Youngs' modulus (used in VELAS as the ratio of the standard deviation of the elastic modulus to its averages).

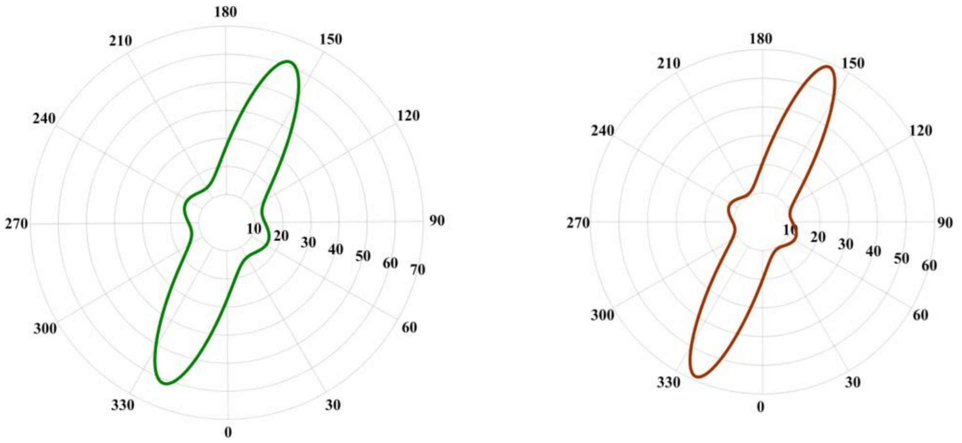

Figure S10. Polar plots of anisotropy of *E* for **1** (green) and **2** (red) in the plane perpendicular to the *a* axis.

5.2. Computed unit cell deformations

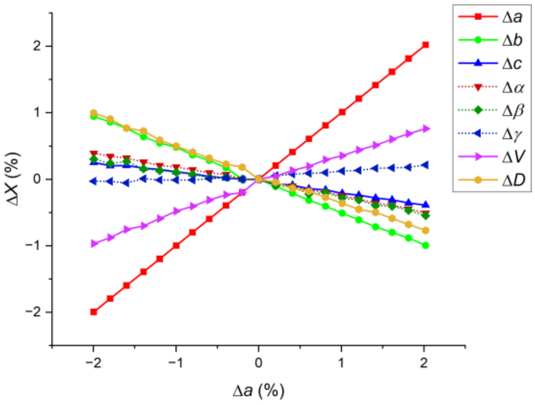

Figure S11. Calculated deformations along the unit cell axes in **1** materializing in response to the applied uniaxial strain in the *a* direction within the range of –2% to +2%.

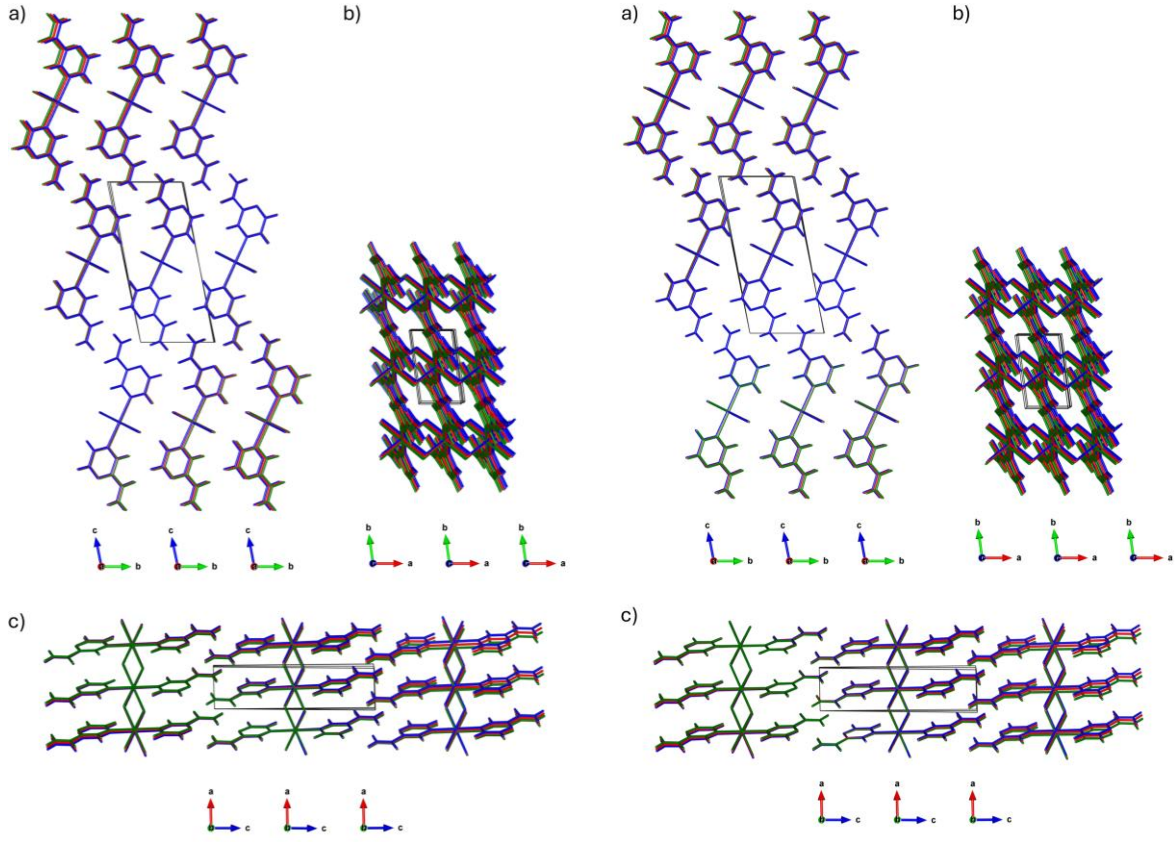

(**1**) (**2**)

Figure S12. Superposition of optimized crystal structures for compounds **1** (left) and **2** (right) under deformation of the crystallographic *a* axis: −2% (blue), 0% (red), and +2% (green). Views along the crystallographic *a*, *b*, and *c* axes.

Table S9. Changes (%) in hydrogen bond geometry obtained on calculated structures for original (no strain) and strained (+/–2%) conditions of materials **1** and **2**.

**1**		**–2% strain**	**no strain**	**+2% strain**
	**Δ*d*(D···A) / %**			
N–H···O		0.0362	0	–0.0724
N–H···N		0.1641	0	–0.0985
C–H···Cl		0.1111	0	–0.2223
	**Δ∠(D–H···A) / %**			
N–H···O		–0.0508	0	0.0282
N–H···N		–0.0856	0	0.0285
C–H···Cl		–0.1625	0	0.1826

**2**		**–2% strain**	**no strain**	**+2% strain**
	**Δ*d*(D···A) / %**			
N–H···O		0.0718	0	–0.0718
N–H···N		0.2915	0	–0.0972
C–H···Br		0.3519	0	–0.2166
	**Δ∠(D–H···A) / %**			
N–H···O		–0.0172	0	–0.0286
N–H···N		–0.2158	0	0.0487
C–H···Br		–0.1019	0	0.0951

Table S10. Fractional atomic coordinates for the unit cell of **1** at three different levels of deformation along the *a* axis, 0%, –2%, +2%.

0% deformation			
$P\bar{1}$			
a = 3.693892 Å	*α* = 101.142822°		
b = 6.406804 Å	*β* = 90.168961°		
c = 14.034226 Å	*γ* = 97.360367°		
Atom	*x*	*y*	*z*
Cd1	-0.500000	-0.500000	-0.500000
Cl2	-0.053023	0.261177	-0.438438
O3	0.071507	-0.468305	0.127537
N4	0.476708	0.277964	0.343206
N5	0.472101	0.061396	0.149914
N6	0.220755	0.265281	0.005631
C7	0.367654	0.366173	0.270381
H8	0.279902	-0.475203	0.289584
C9	0.359578	0.256562	0.174233
C10	-0.419960	-0.025804	0.223633
H11	-0.329130	-0.184006	0.205211
C12	-0.420780	0.080100	0.320556
H13	-0.334150	0.009505	0.380310
C14	0.210212	0.360394	0.098632
H15	0.348270	0.131865	-0.016758
H16	0.110507	0.337918	-0.046491

–2% deformation			
$P\bar{1}$			
a = 3.620604 Å	*α* = 101.565506°		
b = 6.465450 Å	*β* = 90.553970°		
c = 14.079782 Å	*γ* = 97.297218°		
Atom	*x*	*y*	*z*
Cd1	-0.500000	-0.500000	-0.500000
Cl2	-0.051610	0.260363	-0.438340
O3	0.075099	-0.467182	0.127287
N4	0.475660	0.278649	0.342955
N5	0.469962	0.062552	0.149772
N6	0.220904	0.265492	0.005644
C7	0.369931	0.367166	0.270243
H8	0.285918	-0.474050	0.289544
C9	0.360439	0.257642	0.174114
C10	-0.424764	-0.024761	0.223378
H11	-0.336829	-0.182938	0.204835
C12	-0.425675	0.080869	0.320262
H13	-0.342320	0.010099	0.379998
C14	0.212291	0.361265	0.098519
H15	0.347385	0.131671	-0.016585
H16	0.109951	0.337559	-0.046453

+2% deformation			
$P\bar{1}$			
a = 3.768267 Å	*α* = 100.655159°		
b = 6.346267 Å	*β* = 89.737450°		
c = 13.982647 Å	*γ* = 97.497078°		
Atom	*x*	*y*	*z*
Cd1	-0.500000	-0.500000	-0.500000
Cl2	-0.055082	0.261729	-0.438470
O3	0.068763	-0.469365	0.127751
N4	0.478249	0.277775	0.343505
N5	0.474651	0.060611	0.150135
N6	0.220419	0.264982	0.005609
C7	0.365958	0.365485	0.270531
H8	0.274718	-0.476139	0.289590
C9	0.359163	0.255720	0.174379
C10	-0.414747	-0.026266	0.224011
H11	-0.321097	-0.184380	0.205745
C12	-0.415427	0.079970	0.320958
H13	-0.325609	0.009717	0.380759
C14	0.208555	0.359595	0.098733
H15	0.348478	0.131912	-0.016890
H16	0.110834	0.338128	-0.046565

Table S11. Fractional atomic coordinates for the unit cell of **2** at three different levels of deformation along the *a* axis, 0%, –2%, +2%.

0% deformation			
$P\bar{1}$			
a = 3.814590 Å	*α* = 100.963593°		
b = 6.564510 Å	*β* = 89.732124°		
c = 13.984485 Å	*γ* = 97.722862°		
Atom	*x*	*y*	*z*
Cd1	-0.500000	-0.500000	-0.500000
Br2	-0.060931	0.254115	-0.435232
O3	0.083486	-0.472525	0.125382
N4	0.486712	0.284484	0.340722
N5	0.469959	0.068369	0.147253
N6	0.219279	0.265396	0.002811
C7	0.377998	0.368262	0.267403
H8	0.293908	-0.476925	0.285938
C9	0.364452	0.259587	0.171335
C10	-0.422662	-0.014642	0.221395
H11	-0.340112	-0.170251	0.203454
C12	-0.416453	0.091229	0.318201
H13	-0.328499	0.023747	0.378024
C14	0.215048	0.360149	0.095990
H15	0.341377	0.134631	-0.019039
H16	0.108007	0.335882	-0.048540

–2% deformation			
$P\bar{1}$			
a = 3.739505 Å	*α* = 101.570526°		
b = 6.615612 Å	*β* = 90.516762°		
c = 14.061633 Å	*γ* = 97.750229°		
Atom	*x*	*y*	*z*
Cd1	-0.500000	-0.500000	-0.500000
Br2	-0.058883	0.253921	-0.435216
O3	0.085249	-0.471901	0.125206
N4	0.482814	0.283704	0.340497
N5	0.467227	0.068939	0.146993
N6	0.220745	0.266108	0.002927
C7	0.377097	0.368074	0.267437
H8	0.295533	-0.477072	0.286245
C9	0.363754	0.259986	0.171292
C10	-0.428417	-0.014644	0.220881
H11	-0.348248	-0.170213	0.202666
C12	-0.423077	0.090596	0.317702
H13	-0.338016	0.022720	0.377392
C14	0.216327	0.360779	0.095985
H15	0.342273	0.135113	-0.018707
H16	0.109252	0.336247	-0.048416

+2% deformation			
$P\bar{1}$			
a = 3.890729 Å	*α* = 100.215858°		
b = 6.513360 Å	*β* = 88.872139°		
c = 13.893723 Å	*γ* = 97.758965°		
Atom	*x*	*y*	*z*
Cd1	-0.500000	-0.500000	-0.500000
Br2	-0.063464	0.253968	-0.434947
O3	0.082598	-0.473141	0.125502
N4	0.491326	0.285829	0.341060
N5	0.473561	0.068307	0.147712
N6	0.217526	0.264561	0.002735
C7	0.379591	0.368789	0.267398
H8	0.292832	-0.476644	0.285560
C9	0.365963	0.259510	0.171453
C10	-0.415894	-0.013860	0.222187
H11	-0.330867	-0.169341	0.204647
C12	-0.408882	0.092671	0.318932
H13	-0.317971	0.025770	0.378941
C14	0.214266	0.359578	0.096008
H15	0.339694	0.133996	-0.019260
H16	0.106260	0.335288	-0.048671

5.2. Calculated interaction energies

Fragments illustrated in Figure S13 are all aligned parallel to crystal faces, fragments (a) to crystal faces (001)/(00$\bar{1}$), and fragments (b) and (c) to the crystal faces (01$\bar{1}$)/(0$\bar{1}$1). However, of the two fragments parallel to the crystal faces (01$\bar{1}$)/(0$\bar{1}$1), only the fragments presented in (b) were considered further as structural changes mainly occur along the weaker links. The interaction energies in (c) are much higher than those in (b), so the fragments illustrated in (c) were excluded from further analysis.

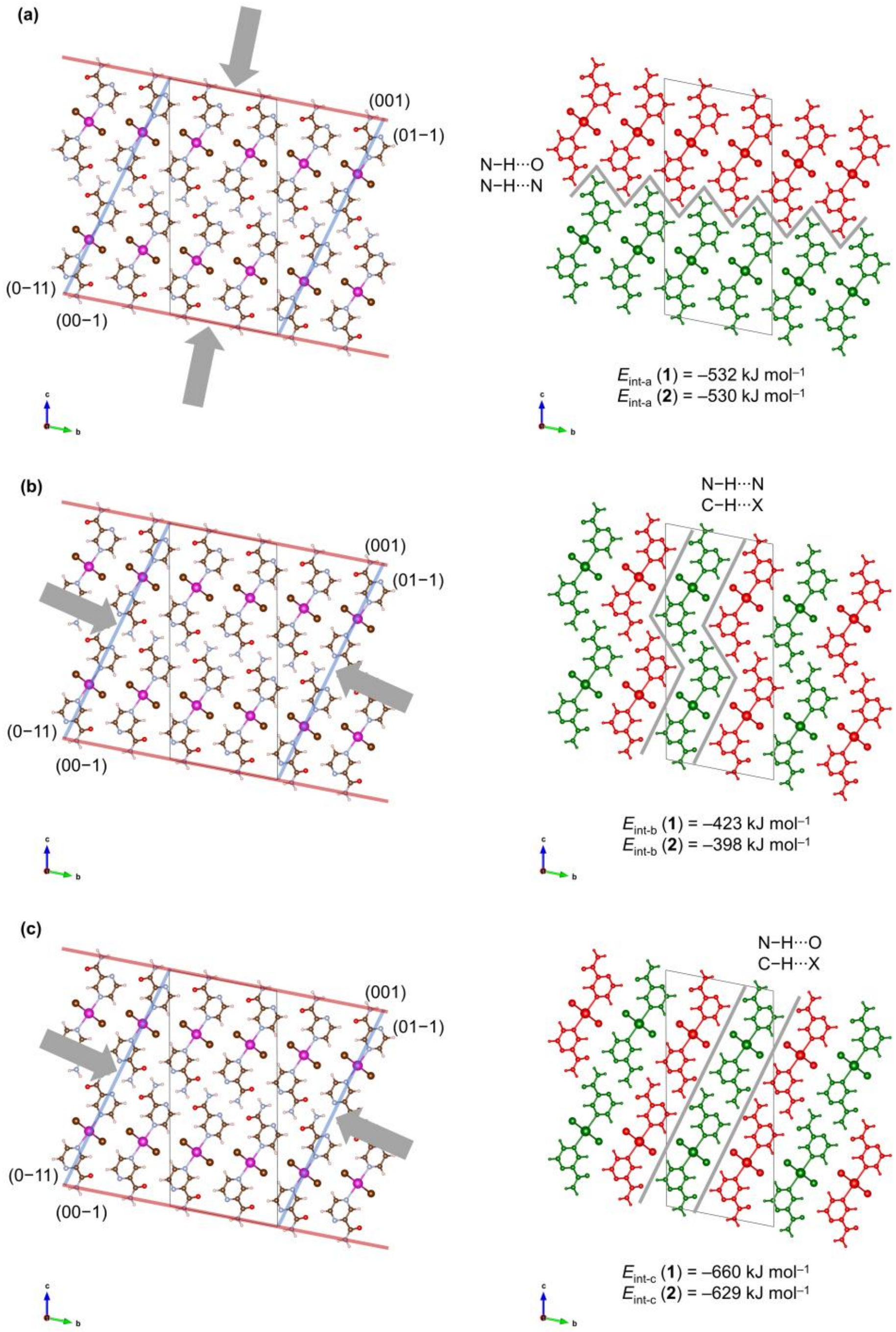

Figure S13. Interaction energies calculated for three interacting molecular pairs (green- and red-colored fragments) within a supercell comprising four 1D coordination polymers. The (a) interaction is parallel to (001)/(00$\bar{1}$) crystal faces, while interactions (b) and (c) primarily describe interactions parallel to (01$\bar{1}$)/(0$\bar{1}$1) crystal faces.

5.3. Calculated Raman spectra

Raman spectra for compounds **1** and **2** were calculated at three different deformation levels: −2%, 0%, and +2%. These levels correspond to compression in the concave part (−2%), an undeformed state (0 %), and extension in the convex region of the crystal (+2%), simulating a total strain of 4%. The results indicate that there are no significant changes in the Raman spectra (Figure S14) for either compound under 2% deformation, which is supportive of the experimental findings (see micro-Raman spectra performed on elastically deformed crystals of compounds **1** and **2**, Figure S16). However, the computational study shows that the peak positions, especially in the high-frequency region, begin to shift, and their intensities exhibit slight variations as the crystal undergoes increased deformation, reaching up to 4% deformation.

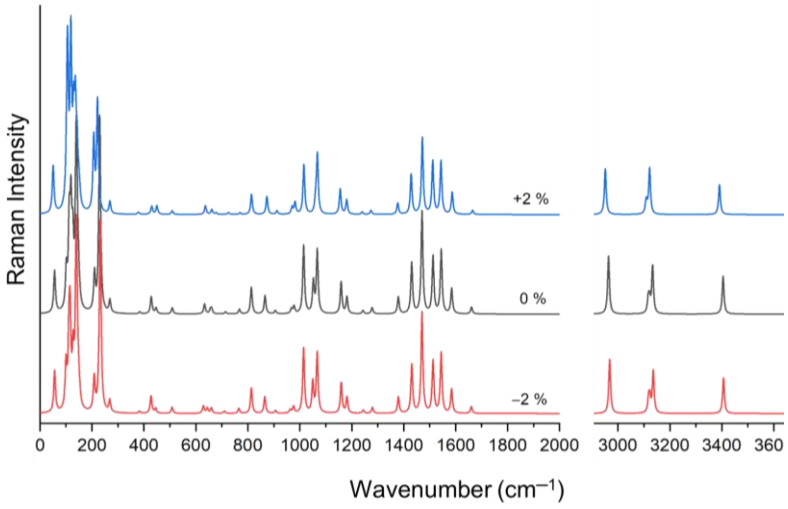

(**1**)

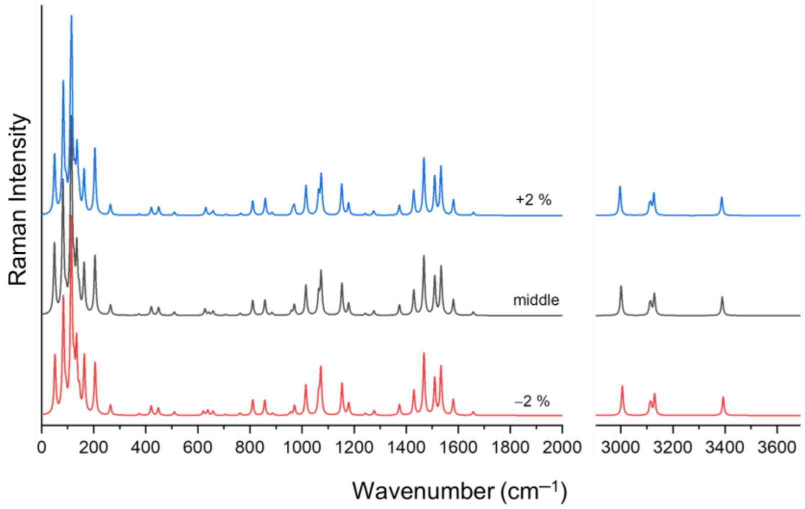

(**2**)

Figure S14. Calculated Raman spectra for **1** (top) and **2** (bottom) under −2%, 0%, and +2% deformations along the crystallographic *a* axis, mimicking compression and extension at the inner and outer arcs, respectively, of the bent crystal.

6. Microfocus Raman spectroscopy

Raman spectra were measured on a Renishaw inVia Raman microscope equipped with a laser emitting at 785 nm using an ×100 objective (NA = 0.85) with a 10 s exposure time and one accumulation. The applied laser power was 3.6 mW. The raw spectra were processed using the WiRE 5.3 software.

Crystal samples for Raman measurements were securely positioned on an aluminium holder, with both crystals' ends affixed using adhesive. This ensured that the crystals remained in a bent configuration throughout the duration of the measurements. The profiling of the crystals was conducted at the point of maximum curvature, and spectra were collected at regular intervals (Figure S15). For each sample, a total of five spectra were recorded, facilitating a thorough analysis. No detectable shifts in peak positions or changes in intensities were observed in the spectra of either compound **1** or compound **2**. Additionally, the calculated Raman spectra corroborated this observation, as no significant changes were noted, even at a deformation of 2% (see Figure S14).

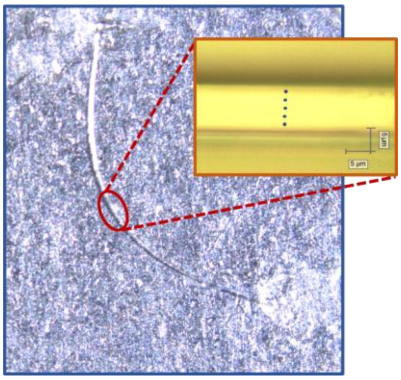

Figure S15. Crystal of **1** fixed on the aluminium holder in a bent shape.

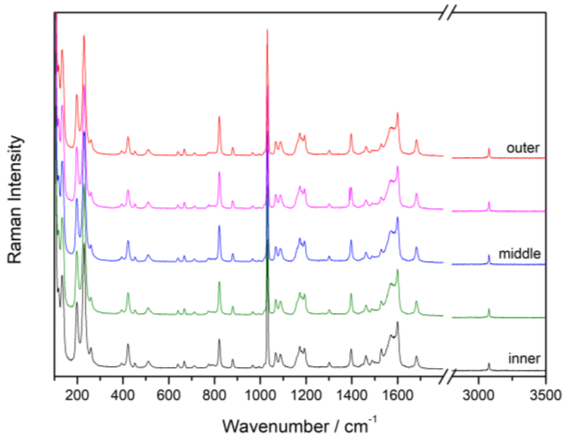

(**1**)

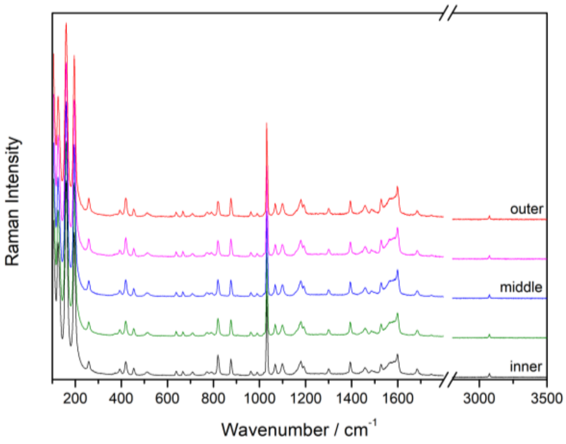

(**2**)

Figure S16. Raman spectra of **1** (left) and **2** (right).

7. Crystal bending experiments

Mechanical responses of **1** and **2** to applied external force were investigated via a modified three-point bending procedure and recorded using DinoLite Microscope camera. Crystals of needle-like morphology were carefully selected under an optical microscope and immersed in a small amount of paratone oil placed on a microscope slide (to prevent crystal damage by the metal accessories). The force was applied in a controlled fashion, at a constant velocity of 100 μm/s, to both major, $(01\bar{1})/(0\bar{1}1)$, and minor, $(001)/(00\bar{1})$, crystal faces.

Crystals were supported at two points from one side, while the force was applied from the opposite one in the third point (Figure S17). Crystals were bent carefully several times before the critical radius was exceeded, which caused the crystal to fracture. The results were quantified using Euler-Bernoulli's beam bending theory.[xvi]

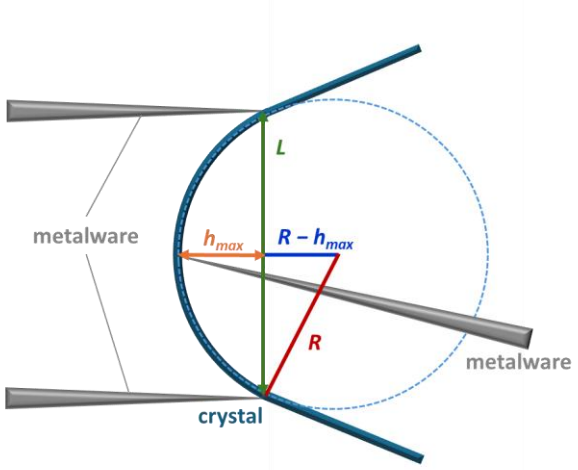

$$R^2 = \left(\frac{L}{2}\right)^2 + (R\text{-}h_{\mathrm{max}})^2 \qquad [1]$$

$$R = \frac{\left(\frac{L}{2}\right)^2 + h_{\mathrm{max}}{}^2}{2h_{\mathrm{max}}} \qquad [2]$$

$$\boldsymbol{\varepsilon}\ (\%) = \frac{\frac{t}{2}}{R} \cdot 100 \qquad [3]$$

Figure S17. Measurement scheme of a bent crystal, including geometrical parameters and equations used to calculate the bending strain (***ε***). Thickness (*t*) of the undeformed crystals, length (*L*), and maximal displacement (h_{max}) used to calculate the radius (*R*) of the bent crystal.

Table S12. Geometrical parameters used to calculate bending strain values (ε) of **1**. The mean values were determined based on twenty different samples, ten for applying the force on the larger crystal faces, $(01\bar{1})/(0\bar{1}1)$ (ε_1; **1-C01** – **1-C10**), and ten for applying the force on the smaller crystal faces, $(001)(00\bar{1})$ (ε_2; **1-C11** – **1-C20**). Thickness (t) measured on the undeformed crystal; length (L) and maximal displacement (h_{max}) measured at the point of maximal curvature (see Figures below).

Sample	$\bar{t}$ / mm	L / mm	h_{max} / mm	R / mm	ε / %
**1-C01**	0.020	2.477	0.665	1.490	0.68
**1-C02**	0.020	2.475	0.796	1.360	0.72
**1-C03**	0.017	1.827	0.333	1.419	0.60
**1-C04**	0.021	1.764	0.218	1.893	0.56
**1-C05**	0.036	2.213	0.226	2.822	0.63
**1-C06**	0.019	1.016	0.097	1.380	0.68
**1-C07**	0.026	1.528	0.129	2.350	0.55
**1-C08**	0.023	1.767	0.183	2.224	0.51
**1-C09**	0.018	1.510	0.203	1.506	0.59
**1-C10**	0.018	2.135	0.542	1.322	0.68
					ε_1 = **0.62 ± 0.07**
**1-C11**	0.039	1.657	0.077	4.496	0.44
**1-C12**	0.063	1.836	0.060	7.053	0.44
**1-C13**	0.036	1.933	0.102	4.630	0.39
**1-C14**	0.053	1.863	0.068	6.414	0.42
**1-C15**	0.077	1.801	0.040	10.156	0.38
**1-C16**	0.047	2.258	0.098	6.552	0.36
**1-C17**	0.038	1.402	0.060	4.125	0.46
**1-C18**	0.039	2.297	0.137	4.883	0.40
**1-C19**	0.038	2.121	0.126	4.526	0.42
**1-C20**	0.061	1.524	0.040	7.278	0.42
					ε_2 = **0.41 ± 0.03**

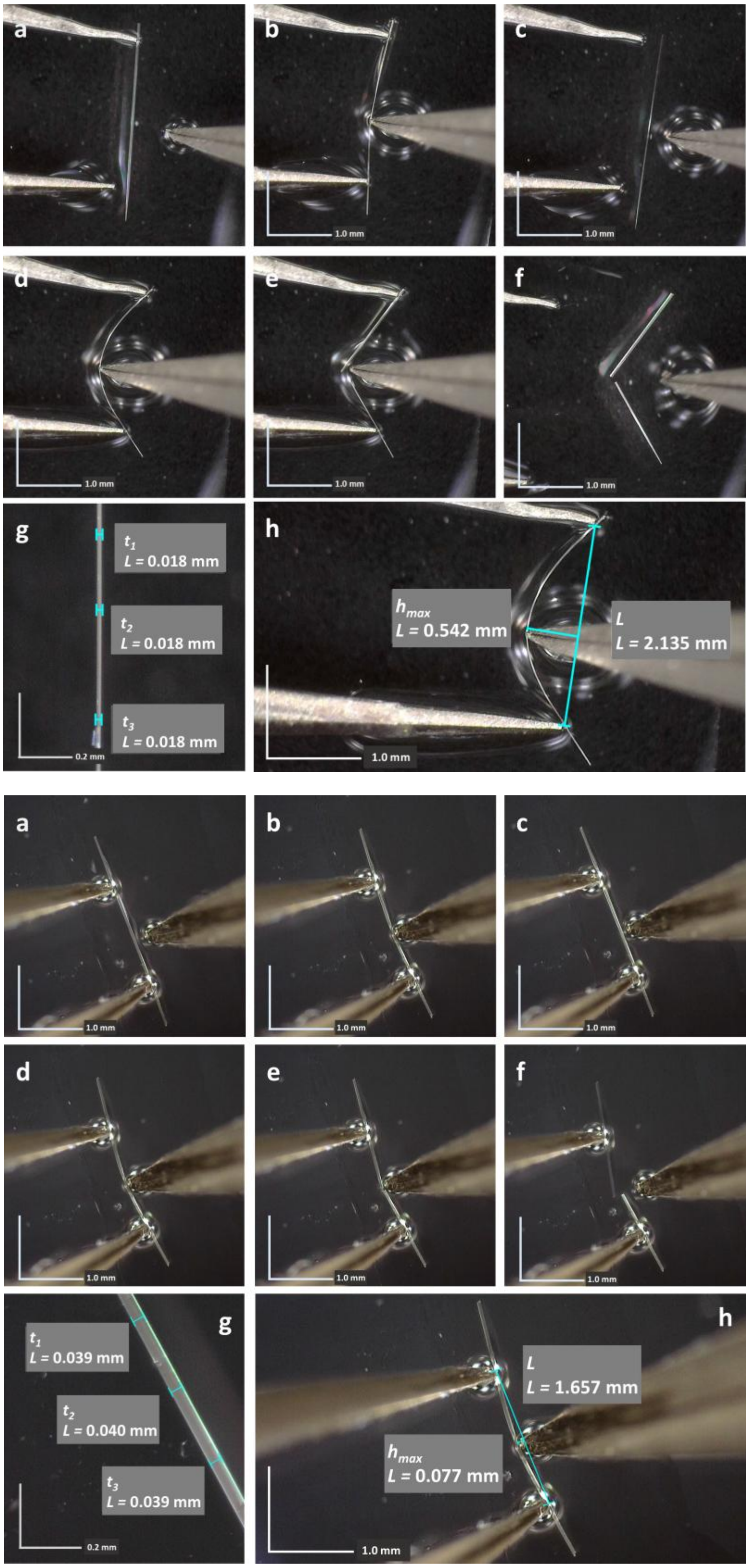

Figure S18. A modified three-point bending experiment of **1**. The force was applied on the larger, (01$\bar{1}$)/(0$\bar{1}$1), top (sample 1-C10; crystal flips: **a**→**b**), and smaller crystal face, (001/(00$\bar{1}$), bottom (sample 1-C11). Crystals in images **a–f** and **h** were magnified 50 times, and in **g** 200 times. Slight elastic bending of the crystal followed by relaxation (**a**–**c**); crystal fractures once bent over the critical radius (**d**–**f**). Geometrical parameters: (**g**) thickness *t*, (**h**) length *L* and maximal displacement *h*.

Table S13. Geometrical parameters used to calculate bending strain values (ε) of **2**. The mean values were determined based on twenty different samples, ten for applying the force on the larger crystal faces, $(01\bar{1})/(0\bar{1}1)$ (ε_1; samples **2-C01** – **2-C10**), and ten for applying the force on the smaller crystal faces, $(001)(00\bar{1})$ (ε_2; **2-C11** – **2-C20**). Thickness (t) measured on the undeformed crystal; length (L) and maximal displacement (h_{max}) measured at the point of maximal curvature (see Figures below).

Sample	$\bar{t}$ / mm	L / mm	h_{max}/ mm	R / mm	ε / %
**2-C01**	0.020	1.495	0.246	1.259	0.79
**2-C02**	0.025	2.259	0.371	1.905	0.66
**2-C03**	0.025	2.329	0.578	1.462	0.84
**2-C04**	0.022	2.532	0.616	1.609	0.69
**2-C05**	0.026	2.348	0.469	1.704	0.75
**2-C06**	0.023	2.123	0.428	1.530	0.74
**2-C07**	0.019	1.897	0.404	1.315	0.72
**2-C08**	0.022	2.215	0.568	1.365	0.79
**2-C09**	0.015	1.647	0.398	1.051	0.70
**2-C10**	0.022	1.959	0.331	1.615	0.67
					**ε_1 = 0.74 ± 0.06**
**2-C11**	0.046	1.742	0.08	4.782	0.48
**2-C12**	0.036	2.08	0.121	4.530	0.39
**2-C13**	0.070	4.100	0.241	8.839	0.40
**2-C14**	0.034	1.876	0.128	3.501	0.49
**2-C15**	0.039	2.378	0.182	3.975	0.49
**2-C16**	0.045	1.713	0.066	5.591	0.40
**2-C17**	0.057	3.475	0.195	7.838	0.37
**2-C18**	0.057	2.112	0.080	7.010	0.40
**2-C19**	0.036	1.534	0.077	3.859	0.46
**2-C20**	0.090	2.732	0.088	10.646	0.42
					**ε_2 = 0.43 ± 0.04**

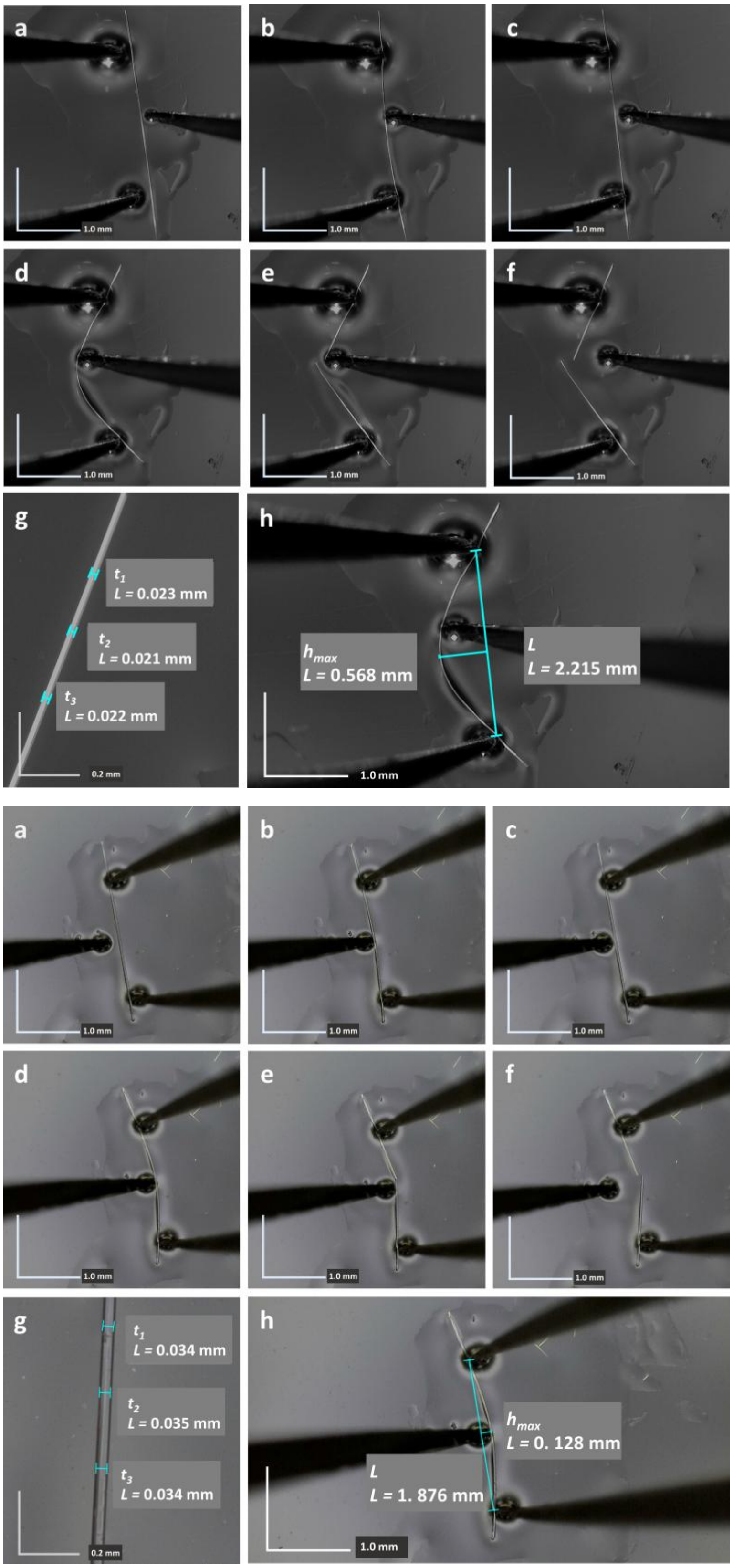

Figure S19. A modified three-point bending experiment of **2**. The force was applied on the larger, $(01\bar{1})/(0\bar{1}1)$, top (sample 2-C08), and smaller crystal face, $(001/(00\bar{1})$, bottom (sample 2-C14). Crystals in images **a–f** and **h** were magnified 50 times, and in **g** 200 times. Slight elastic bending of the crystal followed by relaxation (**a**–**c**); crystal fractures once bent over the critical radius (**d**–**f**). Geometrical parameters: (**g**) thickness *t*, (**h**) length *L* and maximal displacement *h*.

8. AFM measurements

AFM mechanical measurements were performed at room temperature on a commercial MultiMode 8 AFM (Bruker). NCHV-A silicon probes (Bruker, resonance frequency 320 kHz, spring constant 42 N m^{-1}) were used to determine the mechanical properties of the straight crystals **1** and **2**. The deflection sensitivity of the cantilever was estimated from the slope of the loading curve obtained on the sapphire sample and the spring constant was calibrated according to the Sader method.[xvii] Reference measurements on a polystyrene standard sample (PSFILM-12M, Bruker, E = 2.70 GPa, v = 0.34) were used to estimate the tip radius (the nominal tip radius is 8 nm and estimated tip radius values were in the range from 16 nm to 19 nm).

Freshly prepared crystals were extracted from the solution and glued on the metal disks in such a way that the examined crystal lies on a facet of larger dimension ($01\bar{1}/0\bar{1}1$). Force-separation curves were acquired using NanoScope software v9.7 in the force volume mode, where the matrix of 16 x 16 force curves was obtained on the crystal surface of the larger crystal facet ($01\bar{1}/0\bar{1}1$). To ensure the reproducibility and the statistical significance of the results the measurements were conducted on a few different crystal samples of each compound (**1** and **2**). Data analyses were performed using NanoScope Analysis v2.0 (Bruker). Young's moduli of samples were estimated by fitting individual force–separation curves on the Hertzian model (R^2 were approx. 0.95).[xviii] Young's moduli of straight crystals **1** and **2** shown in Table S11 are averaged values of Young's moduli acquired from fitting force-separation curves (at least 3600 data points were collected) for each compound.

Table S14. Young's moduli obtained on straight crystals of **1** and **2** probed on the major crystal faces ($0\bar{1}1/01\bar{1}$).

Compound	1	2
**Young modulus / GPa**	6.33 ± 1.08	5.03 ± 0.80

References

[i] CrysAlisPRO; Agilent Technologies Ltd, Yarnton, England, 2014.

[ii] Dolomanov, O. V.; Bourhis, L. J.; Gildea, R. J.; Howard, J. A. K.; Puschmann, H. OLEX2: A Complete Structure Solution, Refinement and Analysis Program. *J. Appl. Cryst.,* **2009**, *42* (2), 339−341.

[iii] Sheldrick, G. M. SHELXT − Integrated Space-Group and Crystal-Structure Determination. *Acta Crystallogr.,* **2015**, *A71* (1)3−8.

[iv] Sheldrick, G. M. A Short History of ShelX. *Acta Crystallogr*., **2008**, *A64* (1), 112−122.

[v] Macrae, C. F.; Sovago, I.; Cottrell, S. J.; Galek, P. T. A.; McCabe, P.; Pidcock, E.; Platings, M.; Shields, G. P.; Stevens, J. S.; Towler M.; Wood, P. A. Mercury 4.0: From Visualization to Analysis, Design and Prediction. *J. Appl. Cryst.*, **2020**, *53*(1) 226−235.

[vi] Lommerse, J. P. M.; Stone, A. J.; Taylor, R.; Allen, F. H. The Nature and Geometry of Intermolecular Interactions between Halogens and Oxygen or Nitrogen. *J. Am. Chem. Soc.*, **1996**, *118* (13)3108−3116.

[vii] Wojdyla, J. A.; Kaminski, J. W.; Panepucci, E.; Ebner, S.; Wang, X.; Gabadinho, J.; Wang, M.; DA+ Data Acquisition and Analysis Software at the Swiss Light Source Macromolecular Crystallography Beamlines. *J. Synchr. Rad.*, **2018**, *25* (1) 293−303.

[viii] Dovesi, R.; Erba, A.; Orlando, R.; Zicovich-Wilson, C. M.; Civalleri, B.; Maschio, L.; Rérat, M.; Casassa, S.; Baima, J.; Salustro, S.; Kirtman, B. Quantum-Mechanical Condensed Matter Simulations with CRYSTAL. *WIREs Comput. Mol. Sci.* **2018**, *8* (4), e1360.

[ix] Perdew, J. P.; Chevary, J. A.; Vosko, S. H.; Jackson, K. A.; Pederson, M. R.; Singh, D. J.; Fiolhais, C. Atoms, Molecules, Solids, and Surfaces: Applications of the Generalized Gradient Approximation for Exchange and Correlation. *Phys. Rev. B* **1992**, *46* (11), 6671−6687.

[x] Grimme, S.; Antony, J.; Ehrlich, S.; Krieg, H. A Consistent and Accurate ab Initio Parametrization of Density Functional Dispersion Correction (DFT-D) for the 94 Elements H-Pu. *J. Chem. Phys.* **2010**, *132* (15), 154104.

[xi] Vilela Oliveira, D.; Laun, J.; Peintinger, M. F.; Bredow, T. BSSE-Correction Scheme for Consistent Gaussian Basis Sets of Double- and Triple-Zeta Valence with Polarization Quality for Solid- State Calculations. *J. Comput. Chem.* **2019**, *40* (27), 2364−2376.

[xii] Momma, K.; Izumi, F. VESTA 3 for Three-Dimensional Visualization of Crystal, Volumetric and Morphology Data. *J. Appl. Crystallogr*. **2011**, *44* (6), 1272−1276.

[xiii] Boys, S. F.; Bernardi, F. The Calculation of Small Molecular Interactions by the Differences of Separate Total Energies. Some Procedures with Reduced Errors. *Mol. Phys.* **1970**, *19* (4), 553−566.

[xiv] Ran, Z.; Zou, C. M.; Wei, Z. J.; Wang, H. VELAS: An Open-source Toolbox for Visualization and Analysis of Elastic Anisotropy. *Comp. Phys. Commun*. **2023**, *283*, 108540.

[xv] Ranganathan, S.I.; Ostoja-Starzewski, M. Universal Elastic Anisotropy Index. *Physical Review Letters* **2008**, *101* 055504.

[xvi] Timoshenko, S. Strength of materials, D. Van Nostrand Company, New York, 1940.

[xvii] Sader, J. E.; Chon, J. W. M.; Mulvaney, P. Calibration of Rectangular Atomic Force Microscope Cantilevers. *Rev. Sci. Instrum.*, **1999**, *70* (10), 3967−3969.

[xviii] Hertz, H. J. Ueber die Berührung fester elasticher Körper. *Reine Angew. Math.*, **1881**, *92*, 156−171.
